# Reliability of the serial reaction time task: If at first you don’t succeed, try, try, try again

**DOI:** 10.1177/17470218241232347

**Published:** 2024-03-07

**Authors:** Cátia M Oliveira, Marianna E Hayiou-Thomas, Lisa M Henderson

**Affiliations:** Department of Psychology, University of York, York, UK

**Keywords:** Language, serial reaction time task, procedural memory, sequence learning, reliability, individual differences

## Abstract

Procedural memory is involved in the acquisition and control of skills and habits that underlie rule and procedural learning, including the acquisition of grammar and phonology. The serial reaction time task (SRTT), commonly used to assess procedural learning, has been shown to have poor stability (test–retest reliability). We investigated factors that may affect the stability of the SRTT in adults. Experiment 1 examined whether the similarity of sequences learned in two sessions would impact stability: test–retest correlations were low regardless of sequence similarity (*r* < .31). Experiment 2 added a third session to examine whether individual differences in learning would stabilise with further training. There was a small (but nonsignificant) improvement in stability for later sessions (Sessions 1 and 2: *r* = .42; Sessions 2 and 3: *r* = .60). Stability of procedural learning on the SRTT remained suboptimal in all conditions, posing a serious obstacle to the use of this task as a sensitive predictor of individual differences and ultimately theoretical advance.

## Introduction

Procedural memory underlies the encoding; storage; and retrieval of motor, perceptual, and cognitive skills that involve the integration of sequenced, statistical, and probabilistic knowledge across the lifespan (Eichenbaum, 2002; Eichenbaum & Cohen, 2001; Koch et al., 2020; Ullman, 2004). Learning in this system relies on the basal ganglia (specifically, the striatum), the cerebellum, and portions of the parietal and frontal cortices (Packard & Knowlton, 2002; Parent & Hazrati, 1995; Poldrack & Packard, 2003) and tends to be gradual, yet once the skills have been learned they are used rapidly and automatically. The procedural memory system is proposed to be involved in language acquisition. Specifically, Ullman and colleagues (Ullman, 2004; Ullman et al., 2020) propose that the procedural memory system supports the acquisition of rule-based linguistic knowledge, such as phonology and grammar; while the declarative system is mostly associated with acquisition of more arbitrary and explicit knowledge, such as vocabulary. Supporting this, language and procedural memory share brain systems, including basal ganglia and frontal cortex, especially Broca’s area (Ullman, 2001; Ullman & Pierpont, 2005), and clinical populations with impairments of the basal ganglia tend to show both motor and linguistic impairments (Ullman & Pierpont, 2005). Aligning with the declarative/procedural model, some previous studies have shown small to moderate correlations between procedural learning and language and literacy abilities (Clark & Lum, 2017; Desmottes et al., 2017; Lum et al., 2012). However, other studies have failed to replicate these associations (Desmottes et al., 2017; Gabriel et al., 2015; Henderson & Warmington, 2017; Siegelman & Frost, 2015; Vakil et al., 2015; West et al., 2019). This inconsistency, coupled with recent concerns about the psychometric properties of tasks used to measure procedural learning—serial reaction time task (SRTT; Kalra et al., 2019; Siegelman & Frost, 2015; Stark-Inbar et al., 2017; West et al., 2018); contextual cueing and Hebb tasks (West et al., 2018); and statistical learning tasks (Arnon, 2020)—calls for further research to systematically examine the reliability of markers of procedural learning.

The SRTT (Nissen & Bullemer, 1987) is the most widely used measure of procedural (or sequence) learning that requires participants to connect a series of events and form high-order associations to predict future positions (Keele et al., 2003). It has been shown to rely on the same neural networks as other measures of procedural learning (Clark et al., 2014; Hardwick et al., 2013). For example, patients with basal ganglia disorders (e.g., Huntington’s disease) show impaired procedural learning on the SRTT (Willingham & Koroshetz, 1993), and functional magnetic resonance imaging (fMRI) studies demonstrate that procedural learning captured by the SRTT elicits activation in the basal ganglia (putamen: Willingham et al., 2002; ventral striatum: Doyon et al., 1996; and the cerebellum: Hardwick et al., 2013). In the SRTT, a stimulus is presented in an array (e.g., four squares presented horizontally across a screen) and participants are required to press a corresponding button on a keypad or button box to the position of the stimulus on screen as quickly as possible. Unbeknown to the participant, some of the stimulus transitions follow a sequence, with procedural learning being measured as the response time difference between the sequenced and random trials. Faster responses to sequenced than random trials are taken as a “procedural learning effect,” indicating that the participant has learned the sequence and is therefore able to anticipate the next position.

SRTTs can be deterministic or probabilistic. Deterministic sequences usually comprise random and sequenced blocks. The first blocks typically contain the repeating sequence, with a sudden switch to a random block, followed by a final sequenced block; however the opposite pattern (random–structured–random) is also frequently adopted. Reaction times (RTs) tend to decrease progressively during practice in sequenced blocks but then increase in random blocks; this difference in RT is taken as evidence of procedural learning. In contrast, probabilistic SRTTs usually comprise two second-order conditional sequences, one that occurs with a higher probability than the other (e.g., sequence A [85%]: 121432413423; sequence B [15%]: 323412431421; Siegelman & Frost, 2015). Each block starts with a random bigram (e.g., 43) and the next location selected will be either the location that followed that bigram in sequence A (i.e., 2, termed a “probable” trial) or the location that following that bigram in sequence B (i.e., 1, termed an “improbable” trial). Procedural learning in probabilistic SRTTs is measured as the difference in response times between probable and improbable trials. Importantly, despite participants showing evidence of procedural learning, they often have little to no awareness of the presence of a probabilistic sequence (Destrebecqz & Cleeremans, 2001). Deterministic sequences, on the contrary, have been found to yield more explicit awareness of the sequence (Jiménez & Vázquez, 2005; Stark-Inbar et al., 2017; Stefaniak et al., 2008). Thus, the probabilistic sequences may represent purer measures of implicit procedural learning (Stefaniak et al., 2008).

The SRTT is well known for producing robust effects at the group level, thus recently there has been increased interest in using the SRTT as a marker of individual differences (Siegelman & Frost, 2015). However only a few studies have explored the psychometric properties of the task. Reliability refers to the ability of a task to rank individuals’ performance consistently across time, with higher reliability indicating stable scores obtained at test and retest (Hedge et al., 2018). Split-half reliability, a measure of internal consistency within a single session that reflects the correlation between scores within a test (Nunnally & Bernstein, 1994), has been shown to be moderate to adequate on the SRTT in children and adults, respectively (children: *r*s = .49−.75; adults *r*s = .84−.92, West et al., 2018, 2021). However, test-retest reliability (i.e., the *stability* of the test scores over different sessions) is notably poorer and below acceptable psychometric standards: that is, r < .70 (Burlingame et al., 1995; Nunnally & Bernstein, 1994), in both children (probabilistic SRTT: r = .21, 500 trials, West et al., 2018; r = .26, 1000 trials, West et al., 2021) and adults (deterministic SRTT: r = .38, Kalra et al., 2019; r = .07, Stark-Inbar et al., 2017; probabilistic SRTT: r = .47, Siegelman & Frost, 2015; r = .70, West et al., 2021; and alternating SRTT: r = .46, Stark-Inbar et al., 2017). In one exception, West et al. (2021) obtained a test–retest reliability of .70 using a probabilistic SRTT with 46 adults aged between 18 and 61 years. The unusually high stability reported here could be due to one or more of a number of methodological differences: for example, a large number of trials (i.e., 1,500), the same sequence was administered twice, the gap between tests was 2–3 days, and use of a 250-ms interstimulus interval (ISI).

According to classical test theory (Fleiss, 1986), observed scores reflect true scores and measurement error, and higher degrees of measurement error lead to greater fluctuations in scores across time. This translates into poor test–retest reliability as participants’ relative ranking will change between test and retest (Berchtold, 2016; Nunnally & Bernstein, 1994). Poor reliability may contribute to noisier predictions; increased uncertainty in parameter estimation (Loken & Gelman, 2017); and attenuation of the association between measures (Rouder et al., 2019; Rouder & Haaf, 2019, 2021). In small samples, as demonstrated by Loken and Gelman (2017), measurement error can lead, by chance, to overestimation of the effect size. Thus, the poor reliability of the SRTT may contribute to the inconsistently reported correlations between language/literacy measures and procedural learning (LeBel & Paunonen, 2011). It is, however, important to note that in the one study to date which reports adequate test–retest reliability for the SRTT (*r* = .70; West et al., 2021), only negligible correlations were observed between procedural learning and word and nonword reading measures (*r*s from −.06 to −.11; West et al., 2021). Thus, even in the face of adequate stability, this lack of association remains contrary to the predictions of the declarative/procedural model. Nevertheless, it is a single study, and identifying optimal conditions for achieving better reliability remains imperative. Indeed, only a robust and reliable task can test the boundaries of the procedural/declarative model of language acquisition, including the procedural deficit hypothesis, and permit a better understanding of the role of procedural learning and language development and disorder (Matheson, 2019). Systematically examining the stability of the SRTT also has clear methodological value, in revealing design modifications to enhance its psychometric properties, and clinical value, in working towards developing a tool that can identify procedural learning weaknesses (Berchtold, 2016). Generally, it has been claimed that a larger number of trials in any task tends to increase reliability, due to a reduction in measurement error (D. H. Baker et al., 2021; Rouder & Haaf, 2019, 2020). However, studies by West and colleagues (2018, 2021) showed only modest (and nonsignificant) numerical improvements in test–retest reliability when they increased the number of trials in their SRTT.

In addition to examining reliability, agreement, also called repeatability, was examined using the Bland–Altman method (Bland & Altman, 1986, 1999, 2010). As argued by Berchtold (2016), the concept of test–retest refers to both the reliability and agreement of a measurement tool, with agreement referring to the ability of a test to produce the same scores when participants are tested under the same conditions. Thus, while reliability reflects the test’s ability to rank participants consistently within or across sessions, agreement instead focuses on the consistency of the scores, independently of the range and distribution of the variables. Thus proving particularly important for clinical applications whereby participants’ scores, instead of ranking order, may be used to track response to intervention.

Therefore, here, we examine further factors that may influence stability. Of particular focus here are the similarity of the sequences to be learned (Experiment 1) and the number of sessions across which learning is assessed (Experiment 2). To allow for a comprehensive understanding of reliability, a multi-measurement analytic approach will be taken: we will assess the psychometric properties of the SRTT across different measures of procedural learning (difference scores or random slopes) and different psychometric measures (split-half reliability, test–retest reliability, and agreement).

## Experiment 1

There are several reasons why the similarity of sequences to be learned over two or more sessions may influence both the size of the procedural learning effect and potentially also its stability, and each predicts that greater similarity between sequences should result in better learning at later sessions. First, learning the same or similar sequences reduces the likelihood of proactive interference, in which the memory of the first-learned sequence disrupts the learning of the second-learned sequence (Borragán et al., 2015; Darby & Sloutsky, 2015). Second, greater similarity increases the likelihood that consolidation of the first sequence will benefit learning of the second, such that individuals benefit from prior knowledge when exposed to the new material (Nemeth et al., 2010; Robertson et al., 2004; Siegelman & Frost, 2015). Third, the well-established phenomenon of practice effects is likely to lead to an improvement in performance for later sessions (Hausknecht et al., 2007; Scharfen et al., 2018), which is why the use of alternate forms is generally recommended (Beglinger et al., 2005); although see Scharfen et al. (2018) for evidence that alternate forms do not reduce practice effects in working memory capacity tasks. Finally, greater similarity may also lead to increased explicit awareness of the sequence at subsequent sessions and improve performance (Rüsseler et al., 2003) as explicit knowledge has been shown to increase with extended training in the SRTT and is more likely to lead to offline consolidation (Robertson et al., 2004).

While greater similarity in sequences used in different sessions may result in larger procedural learning effects in later sessions, they may also reduce the stability of procedural learning (Stark-Inbar et al., 2017). Individual differences in any one of the above factors would introduce variability in procedural learning at retest, thus leading to changes in the rank order of scores (Hedge et al., 2018; Stark-Inbar et al., 2017). Practice effects have been shown to vary according to participants’ characteristics (e.g., age: Brown et al., 2009; Hodel et al., 2014) and cognitive skills (Schaefer & Duff, 2017), thus introducing additional variability at retest. To our knowledge there has been no direct examination of the effect of sequence similarity on either the magnitude of the procedural learning effect, or the test–retest reliability of the SRTT. However, two recent studies in the literature are consistent with our prediction: Siegelman & Frost (2015) used the same sequences at both testing sessions and reported lower test–retest reliability than West et al. (2021), who used different sequences. While West et al. (2021) showed no significant differences in the learning effect between sessions, Siegelman and Frost (2015), on the contrary, reported that after 3 months the majority of participants (64 out of 75) showed a better performance at retest.

Experiment 1 examined the effect of similarity of the two sequences to be learned, to ascertain (1) the impact on the magnitude of the procedural learning effect, and (2) the effect on test–retest reliability (referred to here as stability). Similarity was operationalised in terms of the Levenshtein distance (LD), which has been widely used to determine the distance between strings across fields such as biology, computer science, and linguistics (Berger et al., 2021; Eriksen & Tougaard, 2006; Faes et al., 2016; Konstantinidis, 2005). Three types of operations are considered—substitutions, deletions, and insertions—with a small distance between sequences indicating higher similarity and a large distance revealing that the sequences are dissimilar (Levenshtein, 1966). We used sequences of varying similarity in a probabilistic SRTT to test four main hypotheses:

H1: Participants will demonstrate procedural learning in both sessions, as indexed by faster responses to probable versus improbable elements of the sequence;
H2: Similarity between sequences will impact the magnitude of the procedural learning. Higher levels of similarity between Sessions 1 and 2 will result in a larger procedural learning effect in Session 2, whereas lower levels of similarity between Sessions 1 and 2 will result in a relatively smaller of procedural learning effect;
H3: Within session reliability (indexed by the split-half correlation coefficient) will be higher than stability across sessions, indexed by test–retest reliability;
H4: Sequence similarity will be negatively associated with stability: more similar sequences at Sessions 1 and 2 will be associated with lower test–retest reliability.

### Methods

#### Participants

A total of 103 undergraduate students from the University of York (91 females), aged between 18 and 25 years (*M* = 19.18, *SD* = 1.09), participated in exchange for course credit. The sample included monolingual, bilingual, and multilingual individuals from various nationalities; all identified as fluent English speakers. The sample size was determined based on West et al. (2021), doubling the number of participants to allow for a median split of participants based on similarity of the sequence. Sensitivity analyses, in line with those conducted by Farkas et al. (2023) and presented in Supplementary Materials 1, suggest that sample sizes above 100 participants offer limited gains in precision. Furthermore, with a sample size of 103, we have 80% power to detect correlations equal to, or above, .30. The experiment was approved by the Ethics Committee of the Psychology Department at the University of York and each participant gave written informed consent.

#### Measures

*SRTT*: A nonverbal probabilistic SRTT was used, following West et al. (2018, 2021) given the task used in this previous study has produced the highest reported stability in the existing literature. On each trial, four black outlined rectangles were presented horizontally and a stimulus (i.e., a smiley face) appeared in one of the four rectangles, with participants asked to respond as quickly and accurately as possible by pressing one of four corresponding keys (Z, X, N, M) on the keyboard. The stimulus remained visible until the key press. Participants rested their index and middle fingers of each hand on the four keys so they were ready to respond.

Two versions of this task were generated, each containing two different underlying second-order conditional 12 item sequences. The first two sequences were taken from Shanks et al. (2003): probable sequence A—314324213412; improbable sequence A—431241321423, while the second sequences were taken from Schvaneveldt and Gomez (1998): probable sequence B—121342314324; improbable sequence B—123413214243. In second-order conditional sequences, each trial can be predicted based on the previous two trials (Schwarb & Schumacher, 2012). For each SRTT, each block started with the consecutive generation of two random digits (e.g., 21), with that bigram then followed by the digit in probable sequence A (e.g., 3) with 90% of probability or followed by the digit in improbable sequence A (e.g., 4) with 10% probability (after West et al., 2018, 2021). After each response a new bigram was created which continuously followed the same principles. See Additional Analyses 1^1^ for a series of simulations manipulating (1) the overall number of trials and (2) the ratio between trials per condition.

The task comprised 1,000 trials per session, as in West et al. (2021), divided into 20 blocks of 50 trials each. Within each block, trials immediately followed the participants’ response, with no ISI. Breaks between blocks comprised a fixation cross presented centrally on screen for a random duration between 8 and 12 s. The stimuli were programmed in *Psychopy* 2 (Peirce et al., 2019); response accuracy and RT (from stimulus onset) were recorded.

##### Sequence similarity

**Figure 1.**
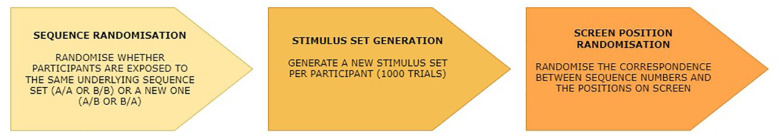
Visual representation of the process of stimulus set generation.

Varying the degree of similarity between inputs was achieved by generating a new stimulus set for each participant (i.e., given the probabilistic nature of the SRTT, each participant was exposed to a different set of 1,000 trials). To achieve variability in the stimulus sets, half of the participants were exposed to stimuli that conformed to the same sequence structure at Sessions 1 and 2 (A/A), while others were exposed to stimuli that were generated by different sequence structures at both time points (A/B) (see Figure 1). Crucially, due to the probabilistic nature of the task, none of the participants was exposed to the exact same stimulus set at both sessions, as new stimuli were generated per session. Furthermore, variability in the input was increased by randomly matching the digits of the sequence (1, 2, 3, 4) to a different position on screen (left, centre–left, centre–right, right). A measure of similarity of the resulting sequences actually presented to each participant was computed using the LD. LD computes the minimum operations required (insertion, deletion, and substitution) for both strings to be identical, thus providing an indication of similarity between stimulus sets (Levenshtein, 1966). The LD was calculated for each participant by comparing the stimulus sets, that is, two sets of 1,000 trials. Across participants, the LD between pairs of stimuli varied between 248 and 437. More details on the distribution of LD between sessions can be found in Additional Analyses 2. The similarity ratio index of the total number of triplets in common between sequences was also computed (Pasquali et al., 2019; Wierzchon et al., 2012). Given the use of second-order conditional sequences, whose minimum unit of sequential information is three sequential locations or triplets, this additional computation ensured that these triplets were captured by the LD scores. Pearson’s correlations between the LD scores and the similarity ratio index revealed a high correlation between measures (*r* = .86).

#### Procedure

All participants were tested individually or in a quiet testing room in groups of up to six. All participants performed the SRTT at both sessions (SRT1 refers to SRTT at Session 1; SRT2 for Session 2). Each session lasted approximately 30 min, with Session 2 occurring 1 week after Session 1 for all but two participants, who were tested 9 and 10 days apart. Once the SRT2 task was completed, task enjoyment and explicit knowledge were assessed via a question and a generation task, to ensure that the levels of explicit awareness were equivalent to previous studies using probabilistic tasks (see Supplementary Materials 2).

#### Statistical analyses

R software—version 4.1.1 (Rstudio Team, 2020) and *lme4* package (Bates et al., 2015) were used to perform two separate linear mixed effects analyses of the performance of the participants on the SRTT and all figures produced using the package *ggplot2* (Wickham, 2016). *p*-values were obtained for the linear mixed effects model using the *lmerTest* package (Kuznetsova et al., 2017) and corrected for multiple comparisons using the Holm–Bonferroni method (Holm, 1979). All reported *p* values are non-adjusted; however, all analyses which have not survived correction for multiple comparisons after correction for familywise error rates have been stated.

For the following data analyses, RTs were grouped into epochs of five blocks, comprising 200 trials. The first two trials of each block were removed as these were not predictable since the sequence follows a higher order structure with the third trial being predicted based on the previous bigram (two trials). All incorrect trials were removed from the analyses. Due to the unequal number of probable and improbable trials, a moving criterion based on sample size was used to identify outlier RTs (Cousineau & Chartier, 2010; Van Selst & Jolicoeur, 1994). Participants with overall RTs > 2.5 *SD* from overall mean were excluded from the analyses (based on *z* scores averaged across probable and improbable conditions for each group/session separately). Two participants were removed from the analyses for both sessions while the remaining two participants were removed for one of the sessions.

As RTs were right-skewed based on visual inspection and tests of normality, a log transformation was used to normalise the distribution of RTs (Brysbaert & Stevens, 2018). Visual inspection of the residual plots after log transformation did not reveal any obvious deviations from homoscedasticity or normality.

The fixed-effects structure represented the maximal-fixed-effects structure. The random intercept structure included solely participants, as item order was not consistent across participants due to randomisation procedures. The random structure followed the forwards best path approach (Barr et al., 2013) starting from the minimal intercepts-only structure and building the random structure according to likelihood-ratio tests (*p* < .2) (Barr et al., 2013) and the Akaike information criterion (AIC; Akaike, 1974) to avoid overfitting (Brewer et al., 2016).

### H1 and H2: the procedural learning effect and similarity

The first model—RT model, designed to explore the procedural learning skills of the sample, included the within-group variables—*probability* (probable or improbable), *epoch* (contrasts between successive Epochs 2-1 [i.e., Epoch 2 vs Epoch 1], 3-2 [i.e., Epoch 3 vs Epoch 2], 4-3 [i.e., Epoch 4 vs Epoch 3], 5-4 [i.e., Epoch 5 vs Epoch 4]), and *Session* (1 or 2) into a linear mixed effects model, with *participants* as a random effect, to account for participant variability in performing the SRTT, and *Session*, *Epoch*, and *Probability* as random slopes. The second model—similarity model—was formulated to explore the relationship between similarity and procedural learning in more detail. Due to the continuous nature of the similarity variable, it was centred and standardised before running the analysis. In both models, the outcome variable is log-transformed RTs to address issues of non-normality, although raw means are reported for ease of interpretation. The model with similarity included only RTs from the last three epochs to avoid the inclusion of epochs where procedural learning is not yet robust as suggested by Conway et al. (2019). *Probability (probable or improbable)*, *Session (1 or 2)*, and *Similarity* were entered as fixed effects and *Participants* as a random effect. Thus, unlike the first model, *Epoch* was not included as the goal was to explore the role of similarity when procedural learning was more robust, independently of its progression across epochs. After building the random structure following the method previously described, *Session* and *Probability* were included as a random slope.

After model selection, the *influence.ME* package was used to detect influential data as these values may lead to changes in regression estimates (Nieuwenhuis et al., 2012). Dfbetas were standardised and participants whose *z*-scores were greater than ±3.29 were identified as influential cases as opposed to the 2.5 *SD* threshold to avoid loss of a high number of participants (Walker et al., 2020). Three participants were identified as influential cases for the response times model and four for the similarity model.

### H3 and H4: reliability and agreement

Test–retest and split-half reliability of the RTs were analysed using Pearson’s correlations, with a reliability of .70 or greater being considered adequate (Nunnally & Bernstein, 1994). Although we have compared our findings against this arbitrary threshold, reliability should be viewed in a continuum. As poor reliability results in the attenuation of the effect sizes of interest, researchers should take these measurement issues into account when designing a study, especially when making design choices, such as the number of trials per individual per task, has a critical impact on the effect sizes within a task and correlations across tasks (Green et al., 2016; Rouder & Haaf, 2019). Two^2^ different indices of procedural learning, commonly used in previous studies, were computed to better capture stability. Simple *difference scores*, the most commonly used measure for the SRTT, were computed for each participant as the simple difference between improbable and probable RTs, with a positive value indicating procedural learning. *Random slopes* for each participant/session were obtained by running a linear mixed effects model with log transformed RTs as a dependent variable and *Probability (probable or improbable)* as a predictor, for the random structure participants were introduced as a random intercept and probability as a random slope (Lammertink et al., 2020; Llompart & Dąbrowska, 2020; Milin et al., 2017). Random slopes were computed as this measure better captures the learning trajectory for each participant and are less likely to be influenced by extreme scores.

To measure split-half reliability for both sessions, trials were separated into probable and improbable trials. Consecutive trials were labelled as odd or even. Split-half reliability was calculated by correlating the overall mean difference in RTs for even and odd trials. The split-half and test–retest reliability were computed both for the entire task and the last 600 trials, following the suggestion that the later stages of procedural learning may be more stable (Conway et al., 2019). Agreement was examined using the Bland–Altman method (Bland & Altman, 1986). The Bland–Altman method involves plotting the mean of the measures for each participant (e.g., (Diff2 + Diff 1)/2 against the difference in the paired measurements in Sessions 2 and 1 (e.g., Diff2—Diff 1), with 95% of the data points being expected to lie within ±1.96 *SD*s of the mean difference, referred to as the 95% limits of agreements. According to Bland and Altman (1999), while a consistent tendency in the scores where performance is superior in one of the sessions than the other can be adjusted for by subtracting the difference between sessions from the one with higher scores (bias), wide limits of agreement pose a more serious problem. Determining whether the limits are adequate will depend on how precise the instrument must be for its use in clinical or research settings.

### Results

Data were available for 100/103 participants for Session 1 and for 98/103 participants for Session 2. Data from five participants were lost due to computer malfunction and one due to a participant being unable to attend the second session. Four of these participants contributed data for one of the sessions, but two participants’ data were lost for both sessions. Three participants were identified as outliers for each session. Data from 97 participants for Session 1 and from 95 participants for Session 2 were therefore included in the analysis. Participants showed high accuracy rates across sessions (Session 1: Macc = 95%, *SD* = .09; Session 2: Macc = 95%, *SD* = .08).

#### H1: procedural learning in the SRTT

**Figure 2.**
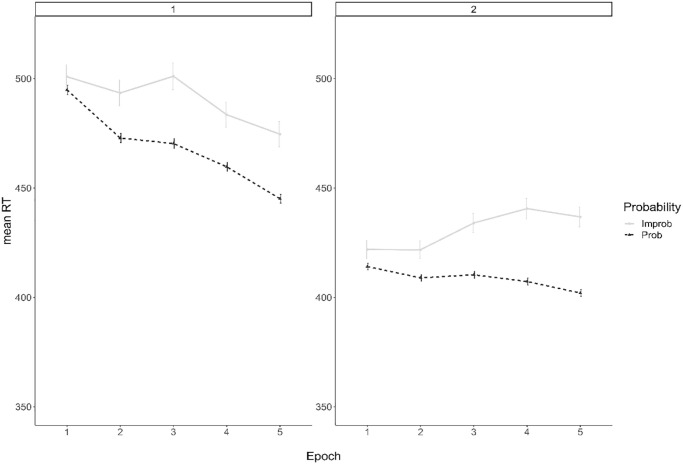
Mean response times for probable and improbable trials per epoch and session (Session 1 on the left and Session 2 on the right). Bars indicate 95% CI.

Results from the mixed effects model are presented in Table 1. As evidenced in Figure 2, RTs decreased with practice as observed by faster RTs with successive epochs. There was evidence of procedural learning, with RTs faster for probable than improbable trials. This “procedural learning effect” increased over epochs, as shown by the significant interaction between *Epoch* × *Probability* for Epoch 2-1 (i.e., Epoch 2 vs Epoch 1), Epoch 3-2 (i.e., Epoch 3 vs Epoch 2), and Epoch 4-3 (i.e., Epoch 4 vs Epoch 3; no longer significant after correction for multiple comparisons), but not for the last contrast, possibly indicating a plateau in learning after Epoch 4. The significant interaction between *Probability* × *Session*, indicates that participants showed a larger procedural learning effect in Session 2 than Session 1, but this was not significant after correction for multiple comparisons. The absence of a three-way interaction between *Epochs × Probability* × *Session* indicates that the within-session progression of procedural learning was similar for both sessions.

**Table 1.** Predictors of the magnitude of procedural learning.

Fixed effects	*b*	*SE*	*t*	*p*	*CI*	
(Intercept)	**6.074**	**0.013**	**474.610**	**<.001**	**6.049**	**6.100**
Epoch 2-1	**−0.019**	**0.004**	**−4.259**	**<.001**	**−0.028**	**−0.010**
Epoch 3-2	0.008	0.004	2.130	.035	0.001	0.015
Epoch 4-3	**−0.010**	**0.003**	**−3.001**	**.003**	**−0.017**	**−0.004**
Epoch 5-4	**−0.019**	**0.004**	**−4.921**	**<.001**	**−0.026**	**−0.011**
Probability	**0.024**	**0.001**	**15.806**	**<.001**	**0.021**	**0.027**
Session	**0.061**	**0.004**	**16.080**	**<.001**	**0.054**	**0.069**
Epoch 2-1 × Probability	**0.011**	**0.002**	**5.020**	**<.001**	**0.007**	**0.016**
Epoch 3-2 × Probability	**0.012**	**0.002**	**5.018**	**<.001**	**0.007**	**0.016**
Epoch 4-3 × Probability	0.005	0.002	2.309	.021	0.001	0.010
Epoch 5-4 × Probability	0.004	0.002	1.653	.098	−0.001	0.008
Epoch 2-1:Session1	**−0.017**	**0.004**	**−3.816**	**<.001**	**−0.025**	**−0.008**
Epoch 3-2:Session1	−0.004	0.003	−1.392	.166	**−**0.010	0.002
Epoch 4-3:Session1	**−0.012**	**0.003**	**−3.398**	**<.001**	**−0.019**	**−0.005**
Epoch 5-4:Session1	**−**0.008	0.003	**−**2.328	.021	**−**0.015	**−**0.001
Probability × Session	**−**0.002	0.001	**−**2.297	.022	**−**0.003	0.000
Epoch 2-1 × Probability × Session	0.001	0.002	0.618	.537	**−**0.003	0.006
Epoch 3-2 × Probability × Session	**−**0.001	0.002	**−**0.396	.692	**−**0.005	0.004
Epoch 4-3 × Probability × Session	**−**0.004	0.002	**−**1.638	.102	**−**0.008	0.001
Epoch 5-4 × Probability × Session	0.000	0.002	0.079	.937	**−**0.004	0.005
Random effects		Variance				*SD*
Participant: (Intercept)		0.0156				0.1250
Participant: Session (Slope)		0.0013				0.0360
Participant: Epoch 2-1 (Slope)		0.0014				0.0372
Participant: Epoch 3-2 (Slope)		0.0008				0.0291
Participant: Epoch 4-3 (Slope)		0.0006				0.0248
Participant: Epoch 5-4 (Slope)		0.0008				0.0286
Participant: Probability (Slope)		0.0002				0.0127
Participant: Session × Epoch 2-1 (Slope)		0.0013				0.0361
Participant: Session × Epoch 3-2 (Slope)		0.0004				0.0203
Participant: Session × Epoch 4-3 (Slope)		0.0006				0.0254
Participant: Session × Epoch 5-4 (Slope)		0.0006				0.0252

Indicated in bold are the contrasts that survived correction for multiple comparisons using the Holm–Bonferroni method.

#### H2: the effect of similarity on procedural learning

**Figure 3.**
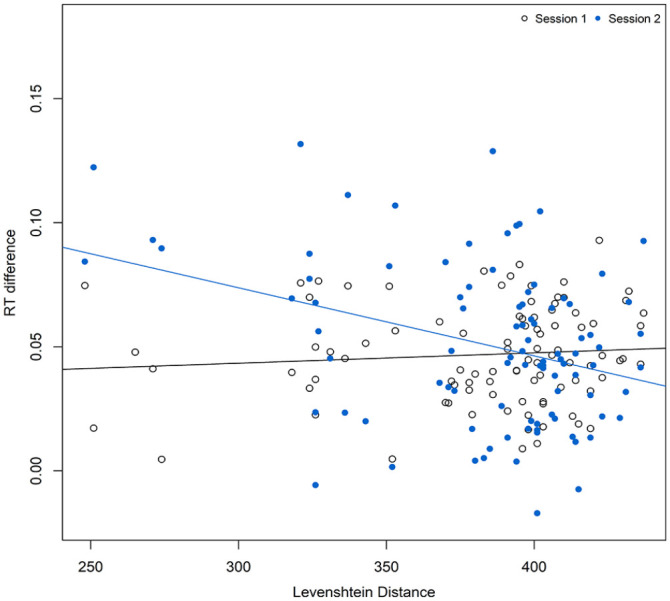
Relationship between Levenshtein distance and difference scores for both sessions for all trials.

In the model incorporating sequence similarity (results are presented in Table 2), a similar pattern of results was obtained in terms of significant effects of *probability* and *session*. Turning to the effect of similarity, in line with our predictions, *LD and LD* × *Probability* were not significant predictors of RT, but there were *Probability × Session × LD* interactions. This indicates that greater similarity was associated with larger procedural learning effects in Session 2. This was further examined by Pearson’s correlations between the LD for each participant and their procedural learning effect (for each session separately). As expected, *LD* and procedural learning were not significantly correlated in Session 1 (given sequence similarity between the two sessions should have no effect on Session 1), overall: *r*(91) = .09, *p* = .40, 95% CI = [−.12, .29]; last 600 trials: *r*(92) = .11, *p* = .271, 95% CI = [−.09, .31], but were moderately negatively correlated in Session 2, overall: *r*(91) = −.34, *p* < .001, 95% CI = [−.51., −.14]; last 600 trials: *r*(91) = −.34, *p* < .001, 95% CI = [−.51, −.15]. This further confirms that participants who were exposed to more similar sequences (i.e., lower *LD*) in Sessions 1 and 2 demonstrated larger procedural learning effects in Session 2 (Figure 3).

**Table 2.** Predictors of the similarity effect on the magnitude of procedural learning.

Fixed effects	*b*	*SE*	*T*	*p*	CI	
(Intercept)	**6.068**	**0.013**	**474.508**	**<.001**	**6.042**	**6.068**
Probability	**0.033**	**0.002**	**19.253**	**<.001**	**0.030**	**0.033**
Session	**0.051**	**0.003**	**15.181**	**<.001**	**0.044**	**0.051**
Levenshtein distance	−0.016	0.015	−1.060	.292	−0.046	−0.016
Probability × Session	−0.002	0.002	−1.524	.131	−0.006	−0.002
Probability × Levenshtein distance	−0.003	0.002	−1.518	.133	−0.007	−0.003
Session × Levenshtein distance	−0.005	0.004	−1.204	.232	−0.013	−0.005
Probability × Session × Levenshtein distance	**0.006**	**0.002**	**3.178**	**.002**	**0.002**	**0.006**
Random effects			Variance			*SD*
Participant (Intercept)	0.0145		0.120			
Participant: Session (Slope)	0.0009		0.030			
Participant: Probability (Slope)	0.0002		0.013			
Participant: Session × Probability (Slope)	0.0001		0.012			

Indicated in bold are the contrasts that survived correction for multiple comparisons using the Holm–Bonferroni method.

#### H3: reliability

Split-half reliability (see Table 3) was very similar in both sessions for the overall task and the last 600 trials; using random slopes rather than raw difference scores as the metric of learning yielded numerically higher estimates of reliability. The split-half coefficients ranged from .55 to .71 (>.70 is considered adequate (Furr & Bacharach, 2008).

**Table 3.** Split-half reliability of the procedural learning measures for overall and last 600 trials of the SRTT for Session 1 (SRT1) and Session 2 (SRT2).

Task	Trials	Split-half reliability			
		*N*	Difference scores	*N*	Random slope
SRT1	1,000	95	*r* = .55 (.39, .67)	95	*r* = .68 (.56, .78)
	Last 600	94	*r* = .50 (.34, .64)	94	*r* = .71 (.59, .80)
SRT2	1,000	91	*r* = .62 (.47, .73)	94	*r* = .70 (.58, .79)
	Last 600	93	*r* = .55 (.39, .68)	93	*r* = .63 (.49, .74)

Split-half reliability correlations are significant (*p* < .05). SRTT: serial reaction time task.

Test–retest reliability of the RTs themselves (e.g., the RT for probable trials in Session 1 with the RT for probable trials in Session 2) was high with a value equal or superior to .80. However, test–retest reliability of procedural learning effect was poor (*r* = .08–.17), irrespective of which measure was used and whether all RTs were included or just the final 600 trials (Table 4).

**Table 4.** Test–retest reliability of the procedural learning measures for overall and last 600 trials of the SRTT.

Task	Trials	Test–retest reliability			
		*N*	Difference scores	*N*	Random slopes
SRT1–SRT2	1,000	91	*r* = .14 (−.06, .34)	91	*r* = .17 (−.04, .36)
	Last 600	91	*r* = .08 (−.12, .28)	91	*r* = .17 (−.04, .36)

Test–retest reliability correlations are nonsignificant (*p* > .05). SRTT: serial reaction time task.

**Figure 4.**
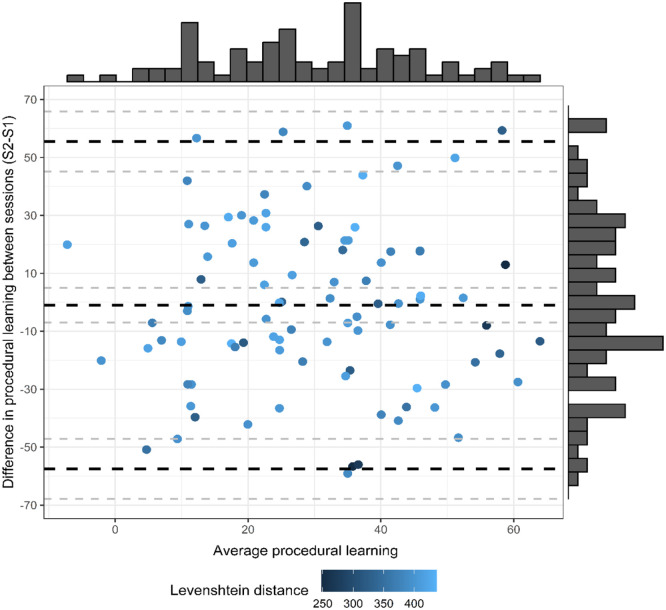
Plot of the procedural learning mean in Session 1 and Session 2 (*x*-axis) against the differences between these measures (*y*-axis). Black dashed line in the centre indicates the overall mean and the blues lines at the top and bottom represent 95% limits of agreement. Grey dashed lines represent CI around each measure.

The levels of agreement between difference scores were explored via creating Bland–Altman plots (Figure 4). The Bland–Altman plots for the difference scores reveal that very few data points lie outside the limits of agreement (−57.53, 55.47), with a mean difference of −1.03; 95% CI = [−7.03; 4.98]. However, although most data points lie within the limits of agreement, there are still considerable discrepancies between time points as evidenced by the poor precision of these limits, indicating a high degree of variance between sessions compared with between-subject variance, thus suggesting that the degree of agreement is not acceptable (Bland & Altman, 1999).

#### H4: similarity and test–retest reliability

Following the significant interaction between similarity and procedural learning, test–retest reliability was compared for participants with low and high sequence similarity scores (achieved by performing a median split). Test–retest reliability was poor for both the high- and low-similarity groups, with no significant differences between groups (overall task: *z* = .83, *p* = .41; last 600 trials: *z* = .15, *p* = .88) (Table 5).

**Table 5.** Test–retest reliability of the procedural learning measures for high and low-similarity groups measured for overall and the last 600 trials of the SRTT.

Similarity	Random slopes		Test–retest reliability
	Trials	*N*	Random slopes
Low	1,000	46	*r* = .30 (.01, .55)
	Last 600	46	*r* = .22 (−.08, .48)
High	1,000	47	*r* = .13 (−.17, .40)
	Last 600	48	*r* = .20 (−.10, .46)

All correlations are nonsignificant (*p* > .05). SRTT: serial reaction time task.

Also, we tested for the possibility that similarity might have an impact on test–retest reliability by including similarity in a linear regression model which predicted the regression slopes in Session 2 from the regression slopes in *Session 1* (mean-centred), *LD* (mean-centred) and their interaction. We found no evidence that *LD* moderated the relationship between procedural learning Sessions 1 and 2. Although *LD* was predictive of the size of the effect in Session 2 (overall: *b* = −.37, *SE* = .11, *t* = −3.55, *p* < .001, 95% CI = [−.58, −.16]; last 600 trials: *b* = −.37, *SE* = .11, *t* = −3.47, *p* < .001, 95% CI = [−.58, −.16]), there was no significant interaction between procedural learning in Session 1 and *LD*, thus suggesting that similarity did not influence the test–retest reliability of the SRTT (overall: *b* = .06, *SE* = .09, *t* = .69, *p* = .493, 95% CI = [−.12, .24]; last 600 trials: *b* = −.006, *SE* = .11, *t* = −.06, *p* = .952, 95% CI = [−.22, .20]).

### Discussion

Experiment 1 examined the reliability of the procedural learning effect, as captured by a probabilistic SRTT, and examined the impact of the similarity of the sequences on the magnitude and stability of procedural learning. As expected, robust procedural learning effects (i.e., faster responses to probable than improbable trials) were observed. However, the level of procedural learning in a subsequent session was substantially influenced by how similar the new sequence was to a previously learned sequence. That is, greater similarity between sequences was associated with larger procedural learning effects for the new sequences. Furthermore, despite observing adequate levels of split-half reliability within each session (random slopes: .68−.72), test–retest reliability was very poor, regardless of the level of similarity between sequences (*r* < .18).

The positive correlation between the procedural learning effect and sequence similarity aligns with previous results (e.g., Siegelman & Frost, 2015). West et al. (2021) tested participants on a probabilistic SRTT, with a 3- to 4-day interval between sessions, and found no significant differences in performance between sessions. However, West et al. used distinct sequences at test and retest with the aim of reducing practice effects. Together with the present results, these studies suggest that the SRTT is prone to practice effects when subsequent sessions use similar sequences. The present study cannot speak of the mechanism/s that underlie the benefit of similarity on procedural learning. However, in light of the lack of evidence for a relationship between explicit awareness and the level of similarity between sequences (see Supplementary Materials 2), one possibility is that consolidated knowledge of the first-learned sequence aids the acquisition of the second-learned sequence (Brown et al., 2009; Press et al., 2005; Robertson et al., 2004) or that knowledge of the first-learned sequence proactively interferes with the acquisition of the second-learned sequence (Desmottes et al., 2017).

The suboptimal test–retest reliability of the SRTT observed here is also generally consistent with previous findings. However, our test–retest coefficients were considerably lower than Siegelman and Frost (2015; *r* = .47) and West et al. (2021; *r* = .70), irrespective of similarity between sequences at both time points. Our coefficients are more akin to those obtained by West et al. (2018, 2021) in children (*r* = .21; *r* = .26, respectively). The low test–retest reliability of the SRTT is striking, particularly in the context of robust group-level procedural learning effects and despite high stability of overall RTs. One possibility is that difference scores, in general, are intrinsically less reliable than their component parts. This has been suggested by Hedge et al. (2018) as difference scores contain measurement error from both measures which leads to an increase in the proportion of measurement error relative to between-subject variance. Yet, the limitations of using difference scores does not seem to pose as much of an issue when analysing the split-half reliability, nor does it explain the better test–retest reliability observed by Siegelman and Frost (2015) and West et al. (2018) despite also analysing difference scores. Furthermore, if difference scores were solely responsible for poor reliability, one would expect better outcomes for the random slopes. Unfortunately, that was not the case. Thus, other factors must contribute to the pattern of lower stability than split-half reliability.

It is possible that specific differences in design between our experiment and West et al. (2021) can account for the divergent findings. First, West et al. (2021) recruited older participants (18–61 years, *M* = 25.33 years, *SD* = 10.33 years) than in Experiment 2 (17–34 years, *M* = 20.09 years, *SD* = 2.09 years). This could have contributed to increasing the stability of the SRTT as test–retest reliability has been found to increase with age in intelligence measures (Schuerger & Witt, 1989). While presentation rates and age of participants have been shown to affect the procedural learning effect on the SRTT (presentation rates: e.g., Arciuli & Simpson, 2011; Emberson et al., 2011; Frensch & Miner, 1994; Soetens et al., 2004; Willingham et al., 1997; age: e.g., Brown et al., 2009; Juhasz et al., 2019) there is no evidence, to our knowledge, of its impact on the test–retest reliability of the task. Second, West et al. (2021) included a 250-ms ISI between trials, which was absent in our experiment with the aim of reducing explicit awareness (Destrebecqz & Cleeremans, 2001). The inclusion of an ISI, however, could have contributed to the higher test–retest reliability by inducing stronger representations of the sequence (Cleeremans & Sarrazin, 2007; Gaillard et al., 2009), with explicit awareness possibly emerging as a consequence of the increased signal strength (Cleeremans, 2011; Timmermans et al., 2012). However, our data did not show indication that the magnitude of procedural learning was associated with explicit awareness (for more details see Supplementary Materials 2). Furthermore, a follow-up experiment (fully described in Supplementary Materials 4) replicated more closely the design adopted by West et al. (2021) by including a 250-ms ISI and participants with ages between 18 and 60 years. Yet, this experiment still revealed suboptimal test–retest reliability (*r* < 21). Explicit awareness levels were also similar between groups with and without an ISI. Taken together, this suggests that the superior reliability observed by West and colleagues (2021) may be explained by other design or sampling factors.

In sum, Experiment 1 obtained clear evidence of procedural learning, which was larger in the second session, particularly when the second-learned sequences were more similar to the first-learned sequences. However, test–retest reliability of procedural learning was very poor regardless of the level of similarity between sequences. Another possibility, examined in Experiment 2, is whether this variability in the procedural learning effect across sessions will diminish with further training—that is, individuals will eventually reach a “plateau” which more accurately reflects their intrinsic procedural learning capacity. Given the lack of evidence for any impact of sequence similarity on reliability of the SRTT, and the larger procedural learning effect for those learning sequences with higher similarity, sequences with high similarity were adopted in Experiment 2 to maximise the chances of participants reaching a “plateau” at an earlier stage of learning.

## Experiment 2

Experiment 2 examined whether the inclusion of three sessions would increase the test–retest reliability of the SRTT, since, as suggested by Conway et al. (2019) the poor reliability of probabilistic procedural learning may be related to the measurement of earlier stages when learning might not be as robust. Palmer et al. (2018) have demonstrated patterns of increased stability on a variety of measures of cognitive ability commonly used to assess striatal dysfunction by increasing the number of training sessions. They reported that practice effects diminished in patients with striatal impairments by the third session, thus increasing the stability of the measures. Although Palmer et al. (2018) did not consider the SRTT, it is possible that it would follow a similar stabilisation trajectory, since the striatum has also been strongly implicated in performance on this task (Robertson et al., 2001; Torriero et al., 2004).

Experiment 2 also carried out a preliminary examination of the relationship between procedural learning and language and literacy. According to the Procedural/Declarative model (Ullman et al., 2020; Ullman & Pierpont, 2005), performance on language measures (particularly grammar and phonology) and literacy measures (e.g., spelling, which requires procedural learning) should be associated with procedural learning. However, such correlations have not been consistently found in previous studies. If these correlations are masked by the low stability of the SRTT and if incorporating multiple sessions increases stability, then stronger correlations would be expected with procedural learning effects measured at later sessions. This hypothesis is supported by West et al. (2021), who found, in their children’s sample, small to moderate correlations between linguistic/literacy measures and procedural learning captured in a second session, but not a first session.

Finally, Experiment 2 considered the role of attention in relation to procedural learning stability. An extensive literature has considered the role of attention in procedural learning in the context of dual task paradigms. Such studies demonstrate a detrimental effect on procedural learning when participants simultaneously perform the SRTT alongside a secondary task (deterministic sequences: Coomans et al., 2014; Schumacher & Schwarb, 2009; Shanks et al., 2003; probabilistic sequences: Shanks et al., 2005). In line with this, a positive correlation between sustained attention and procedural learning in children has been found by Sengottuvel and Rao (2013) and West and colleagues (2021). In the latter, it was also observed that the attentional demands of the SRTT may vary depending on the session: although attention was found to positively correlate with procedural learning at both sessions, stronger correlations were observed for Session 2. Furthermore, when attention was entered as a predictor of children’s attainment (on measures of reading, grammar, and arithmetic), in a latent variable path model which also included the SRTT, measures of declarative learning and attention, attention and declarative memory contributed unique variance, but the SRTT did not. This suggests that while the SRT may be a weak correlate of language and related skills, this may be the result of overlapping variance with other variables, such as attention. This is further supported by the strong correlation between attention and procedural memory (*r* = .56) observed in West et al. (2021).

However, in West et al. (2021), a 9-point observational rating scale was used to estimate the levels of attention throughout the SRTT, while Sengottuvel and Rao (Sengottuvel & Rao, 2013) assessed the offline attention skills through a two-choice RT task. For both attentional tasks information regarding their psychometric properties is lacking, with the operationalisation of attention used by West et al. (2021) potentially tapping into other constructs such as motivation/boredom required for children to remain focused on the task (e.g., R. S. J. d. Baker et al., 2010; Godwin et al., 2016). Here, a direct measure of attention (i.e., a psychomotor vigilance task) was adopted to further explore the relationship between procedural learning and attention.

Experiment 2 used the same SRTT as in Experiment 1 but on three separate sessions, to address three research questions and test the following accompanying preregistered hypotheses (https://osf.io/yb3sv):

H1: Participants are expected to demonstrate evidence of procedural learning in all three sessions.
H2: Moderate to low test–retest reliability levels are expected between Sessions 1 and 2;
H3: If stability of performance increases with the number of sessions, test–retest reliability will be higher between Sessions 2 and 3 than between Sessions 1 and 2;
H4: Split-half reliability will be higher for later sessions when compared with Session 1;
H5: Procedural learning is expected to correlate with language and literacy performance/scores in all sessions;
H6: Higher associations between language and procedural learning will be expected in later sessions if the procedural learning effects are more reliable at later sessions;
H7: Participants with better attention skills will be expected to show more procedural learning;
H8: Higher correlations between procedural learning and attention are expected for later sessions.

No hypotheses were preregistered regarding how attention influences stability between sessions as, to our knowledge, this has not been previously tested using the SRTT. Exploratory analyses were therefore performed to examine relationships between attention and stability.

### Methods

#### Participants

Forty-seven young healthy adults aged between 17 and 34 years (*M* = 20.11 years, *SD* = 2.87 years) with language, literacy, and nonverbal intelligence within the average range (see Supplementary Materials 3) were recruited from the University of York. All participants were native English speakers based in the United Kingdom with normal or corrected-to-normal hearing, vision, and without motor impairments that may impede task performance. Participants received payment or course credit as compensation. The experiment was approved by the Ethics Committee of the Psychology Department in the University of York and each participant gave written informed consent.

#### Measures

##### SRTT

The SRTT used in Experiment 1 was used here, with the exception that the 1,000 trials per session were distributed over 5 blocks rather than 20 to replicate the number of blocks adopted by West et al. (2018, 2021). The first two sequences adopted were the ones included in Experiment 1. A new pair of sequences was selected for the additional session. The sequences were taken from Kaufman et al. (2010): probable sequence E—121432413423; improbable sequence F—323412431421. These sequences were selected to have equivalent levels of similarity (as captured by LD) and the similarity was comparable to West et al. (2018, 2020) (Sequences 1–2: LD = 338; Sequences 1–3: LD = 342; Sequences 2–3: LD = 374).

##### Sustained attention

A computerised 10-min Psychomotor vigilance task (PVT; based on Reifman et al., 2018) was used to measure sustained or vigilant attention by recording response times (RTs) to visual stimuli presented at random intervals between 2 and 10 s ISI. When performing the PVT, participants are asked to press the spacebar as soon as a red counter appears on screen, which stops the counter and displays the RT in milliseconds for a 1-s period. Based on the study by Basner and Dinges (2011), the mean reciprocal response time (*M* 1/RT) was selected as the primary outcome as this measure shows the most superior statistical properties, that is, being sensitive to small changes in fast RTs and robust to extreme values (Basner & Dinges, 2011). Median RTs of the PVT were also adopted as these have shown to have good reliability >.80 in adults (Dorrian et al., 2005).

Beyond these measures on the PVT, performance variability, which may be masked by analyses based on mean performance, has been explored as a valuable source of information to better understand individual differences in learning (Henríquez-Henríquez et al., 2015). The Ex-Gaussian method allows the examination of the response time distribution both for the “mu” and “sigma” parameters of the Gaussian distribution, which represent the mean and standard deviation of the normal component of the distribution, but also “tau,” which represents the exponential component reflecting the slower response times, and is the tail of the distribution. Previous research has found that high indices of intraindividual variability, usually higher tau values, are characteristic of populations with attention-deficit/hyperactivity disorder (ADHD; Borella et al., 2011; Gooch et al., 2012). Thus, the “tau” measure was also computed since it has been proposed as a stronger marker of attention difficulties than basic RTs/lapses (Castellanos et al., 2006). Hence, the “tau” metric would potentially better capture the association between procedural learning and attention.

##### Standardised measures

All cognitive measures were delivered and scored in accordance with manual instructions.

*Nonverbal intelligence* was assessed by the Matrix Reasoning subtest of the Wechsler Abbreviated Scale of Intelligence—Second Edition (WASI-II; test–retest reliability, *r* = .82; Wechsler, 2011). This task consists of 30 incomplete visual matrices and the participants are required to choose the item from a selection of five that correctly completes the matrix.

*Expressive vocabulary* was assessed using the Vocabulary subtest of the Wechsler Abbreviated Scale of Intelligence—Second Edition (WASI-II; test–retest reliability, *r* = .90; Wechsler, 2011). This task requires participants to provide a definition for a series of words that increase in difficulty, presented both verbally and orthographically. Each answer is given a score of 0, 1, or 2 points depending on the quality of the description.

*Nonword repetition* was assessed with the Comprehensive Test of Phonological Processing—2 (CTOPP-2; internal consistency alpha coefficient, *r* = .77; Wagner et al., 2013), providing a measure of phonological memory. Participants were told that they would hear nonwords (that increased in phonological complexity) via headphones and that they should repeat the nonword exactly.

*Sentence repetition* was measured with the Recalling Sentences task from the Clinical Evaluation of Language Fundamentals—Fifth Edition (CELF-5, test–retest reliability, *r* = .94; Wiig et al., 2013) was used to assess individuals’ ability to repeat sentences of increasing length and complexity.

*Reading and spelling* were assessed with the Wechsler Individual Achievement Test, third edition UK (WIAT-III^UK^; internal consistency coefficients *r* ⩾ .90; Wechsler, 2009). For Word Reading, participants were asked to read aloud words and nonwords ordered in increasing difficulty. Participants’ responses were audio-recorded and later scored. The Spelling subtest consists of a spelling-to-dictation task containing regular and irregular words. Participants first heard the target word in isolation, then in the context of a sentence, and finally in isolation again. Dictation was conducted using a recording of a native female speaker.

#### Procedure

A within-subjects design was used, with each participant performing the SRTT at three time points each separated by roughly 1 week (interval between Sessions 1 and 2: *M* = 7.02 days, *SD* = 0.15; interval between Sessions 2 and 3: *M* = 7.07 days, *SD* = .61). The three underlying sequences were counterbalanced across participants and sessions to avoid order effects.

All sessions started with the administration of the SRTT (duration ~15 min). Standardised tests were administered after the SRTT in each session (i.e., literacy and attention tests Session 1; language measures Session 2; nonverbal measure Session 3). A generation task was completed at the end of the final session, to capture explicit knowledge of the sequence learned in Session 3. Session 1 lasted roughly 1 hr; Sessions 2 and 3 were approximately 30 min.

#### Analyses

##### H1: mixed effects model—procedural learning

The same procedures adopted in Experiment 1 were adopted for data treatment and analyses in Experiment 2. The additional session allowed the exploration of its effects on the stability of procedural learning. For the three-level factor of session two orthogonal contrasts were set: lag1 which contrasts Session 1 with Sessions 2 and 3 (S1 vs S2 & S3) and lag2 contrasted the performance in Sessions 2 and 3. After model selection, three participants were identified as influential cases. The analyses reported include the influential cases as this led to no differences in result interpretation with only minor changes in the degree of significance.

##### H2–H4: reliability and agreement

As in Experiment 1, test–retest reliability was calculated between Sessions 1 and 2 and Sessions 2 and 3 using difference scores and random slopes as measures of procedural learning. Agreement was assessed through Bland–Altman plots.

##### H5–H8: relationship between procedural learning and cognitive measures

Pearson’s correlations were conducted to explore the relationship between written and oral language measures and procedural learning. The Holm–Bonferroni method was used to correct for multiple comparisons (Holm, 1979). Based on the sensitivity analysis, this study has 80% power to detect correlations equal and above .35. As nonsignificant results may represent either lack of evidence for a correlation or lack of power, Bayesian Pearson correlations will be computed alongside. Bayes factors above 3 or below ⅓ will be taken as support for the alternative or null, respectively; yet we recognise that Bayes factors should be interpreted in a continuum (Jeffreys, 1961).

##### Exploratory analysis of attention

Ex-Gaussian analysis was performed on the PVT and the parameters were extracted using the package Retimes (Massidda, 2013). The Ex-Gaussian distribution is characterised by a mean mu, standard deviation sigma and exponential distribution with mean tau. In this analysis, we focus on the measure tau as it represents the skewness or variability of the slow responses. This measure has been shown to be a better predictor of performance than traditional response time measures on attention and inhibition tasks (Gooch et al., 2012; Henríquez-Henríquez et al., 2015; van Belle et al., 2015).

### Results

All participants completed the three sessions each separated by 1 week, with the exception that one participant completed Session 3, 11 days after Session 2 and another completed Session 2, 8 days after Session 1. Data from all participants were available for all sessions except for one participant who missed Session 3. The remaining data were included in the analyses. The performance of two other participants was identified as an outlier, one for Session 1 and another for Session 3. Similar to Experiment 1, high levels of accuracy were observed across sessions (Session 1: Macc = 97%, *SD* = .02; Session 2: Macc = 96%, *SD* = .03; Session 3: Macc = 95%, *SD* = .04).

#### H1: procedural learning in the SRTT—effect of session

**Figure 5.**
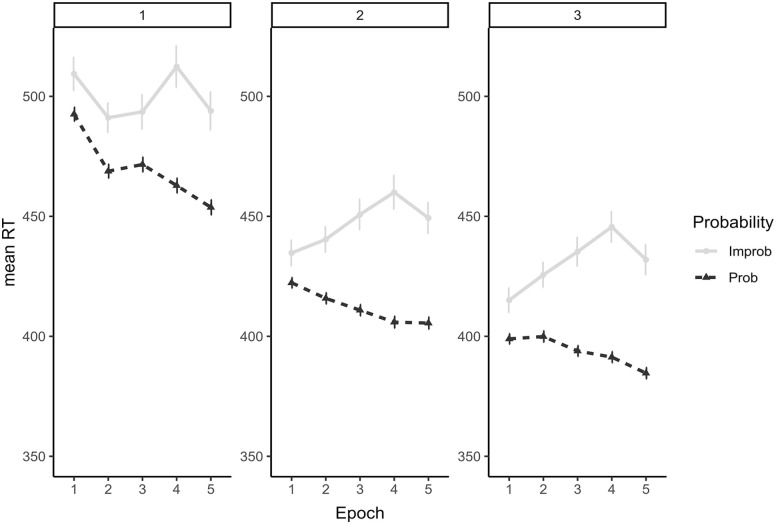
Mean and 95% CI response times for probable and improbable trials per Epoch and Session (Session 1 on the left, Session 2 in the centre, and Session 3 on the right).

Participants’ RTs decreased with practice (Figure 5 and Table 6) as evidenced by significant main effects of *Epoch* for contrasts Epoch 2-1 (no longer significant after correction for multiple comparisons) and Epoch 5-4 and *Session* for both contrasts (Delay1: Session 1 vs Session 2 and 3; Delay2: Session 2 vs Session 3). It is unclear why response times decreased in Epoch 5; however, we hypothesise that fatigue may have contributed to individuals prioritising speed over engaging with the task as demonstrated by the drop in the procedural learning effect (Epoch 5-4 × Probability). Importantly, there was a main effect of *Probability*, as response times were faster for probable than improbable trials. This difference in probable and improbable response times increased with practice as evidenced by a significant *Epoch* × *Probability* interaction (with the exception of the final epoch), as well as a significant *Session × Probability* interaction. Yet, the interaction between *Session × Probability* for Delay2 was no longer significant after correction for multiple comparisons.

**Table 6.** Predictors of the additional session on the magnitude of procedural learning.

Fixed effects	*b*	*SE*	*t*	*p*	CI	
(Intercept)	**6.051**	**0.017**	**347.667**	**.000**	**6.016**	**6.087**
Epoch 2-1	−0.009	0.004	−2.135	.037	−0.018	−0.001
Epoch 3-2	−0.002	0.004	−0.620	.538	−0.010	0.005
Epoch 4-3	0.006	0.004	1.397	.167	−0.002	0.014
Epoch 5-4	**−0.021**	**0.004**	**−5.099**	**.000**	**−0.029**	**−0.012**
Probability	**0.039**	**0.002**	**21.674**	**.000**	**0.035**	**0.043**
Delay1 (S1 vs S2 and S3)	**−0.045**	**0.003**	**−14.568**	**.000**	**−0.051**	**−0.039**
Delay2 (S2 vs S3)	**−0.020**	**0.004**	**−5.271**	**.000**	**−0.028**	**−0.012**
Epoch 2-1 × Probability	**0.014**	**0.003**	**5.438**	**.000**	**0.009**	**0.019**
Epoch 3-2 × Probability	**0.010**	**0.003**	**4.042**	**.000**	**0.005**	**0.015**
Epoch 4-3 × Probability	**0.021**	**0.003**	**8.047**	**.000**	**0.016**	**0.027**
Epoch 5-4 × Probability	−0.008	0.003	−2.763	.006	−0.013	−0.002
Epoch 2-1 × Delay1	**0.013**	**0.002**	**7.406**	**.000**	**0.010**	**0.017**
Epoch 3-2 × Delay1	0.001	0.002	0.828	.408	−0.002	0.005
Epoch 4-3 × Delay1	−0.001	0.002	−0.442	.659	−0.005	0.003
Epoch 5-4 × Delay1	0.003	0.002	1.524	.128	−0.001	0.007
Epoch 2-1 × Delay2	0.004	0.003	1.404	.160	−0.002	0.010
Epoch 3-2 × Delay2	0.003	0.003	0.954	.340	−0.003	0.009
Epoch 4-3 × Delay2	0.001	0.003	0.257	.797	−0.006	0.007
Epoch 5-4 × Delay2	−0.007	0.003	−2.185	.029	−0.014	−0.001
Probability1 × Delay1	**0.004**	**0.001**	**6.757**	**.000**	**0.003**	**0.005**
Probability1 × Delay2	0.003	0.001	2.556	.011	0.001	0.005
Epoch 2-1 × Probability × Delay1	0.001	0.002	0.367	.714	−0.003	0.004
Epoch 3-2 × Probability × Delay1	0.005	0.002	2.644	.008	0.001	0.008
Epoch 4-3 × Probability × Delay1	−0.005	0.002	−2.427	.015	−0.008	−0.001
Epoch 5-4 × Probability × Delay1	0.001	0.002	0.331	.741	−0.003	0.004
Epoch 2-1 × Probability × Delay2	−0.002	0.003	−0.668	.504	−0.008	0.004
Epoch 3-2 × Probability × Delay2	0.002	0.003	0.494	.621	−0.005	0.008
Epoch 4-3 × Probability × Delay2	0.002	0.003	0.516	.606	−0.005	0.008
Epoch 5-4 × Probability × Delay2	−0.001	0.003	−0.403	.687	−0.008	0.005
Random effects		Variance				*SD*
Participant (Intercept)		0.0004				0.113
Participant: Delay1 (Slope)		0.0006				0.019
Participant: Delay2 (Slope)		0.0006				0.024
Participant: Block2-1 (Slope)		0.0004				0.024
Participant: Block3-2 (Slope)		0.0004				0.020
Participant: Block4-3 (Slope)		0.0004				0.020
Participant: Block5-4 (Slope)		0.0001				0.019
Participant: Probability (Slope)		0.0410				0.010

Indicated in bold are the contrasts that survived correction for multiple comparisons using the Holm–Bonferroni method. CI: confidence interval.

Despite this improvement in procedural learning with practice, the three-way interaction between *Epochs × Probability × Session* was only significant for *Delay1* for Epoch 3-2 and Epoch 4-3 (also no longer significant after correction for multiple comparisons), thus indicating a significant increase in procedural learning in Sessions 2 and 3 for Epoch 3-2 relative to Session 1. This difference between Session 1 and Sessions 2/3 for Epoch 3-2 is apparent in Figure 5. The nonsignificant interaction for *Delay2* (Session 2 vs Session 3) indicates that, despite the overall gains in procedural learning from Sessions 2 to 3, the difference between sessions was not observed at the epoch level (Figure 5).

#### H2–H4: reliability

As shown in Tables 7 and 8 and similar to Experiment 1, split-half reliability for the SRTT was numerically higher when using slope coefficients compared with raw difference scores and ranged from low (*r* = .23) to excellent (*r* = .91; Cicchetti, 1994; Cicchetti & Sparrow, 1990). This difference reached significance in the third session for both contrasts (*p* < .001).

**Table 7.** Split-half reliability for the procedural learning measures per session (SRT1, Session 1; SRT2, Session 2; SRT3, Session 3).

Task	Trials	Split-half reliability			
		*N*	Difference scores	*N*	Random slopes
SRT1	1,000	45	.60*** (.38, .76)	45	.77*** (.61, .87)
	Last 600	44	.56*** (.31, .73)	45	.66*** (.45, .80)
SRT2	1,000	45	.55*** (.30, .72)	47	.55*** (.31, .72)
	Last 600	46	.36* (.08, .59)	47	.56*** (.32, .73)
SRT3	1,000	43	.23 (−.07, .50)	44	.81*** (.67, .89)
	Last 600	45	.32 (.02, .56)	45	.91*** (.84, .95)

* *p* < .05. ***p < .001.

**Table 8.** Pairwise test–retest reliability of the procedural learning measures.

Tasks	Trials	Test–retest reliability	
		Difference scores	Random slopes
SRT1–SRT2	1,000	.22 (−.07, .49)	.28 (−.01, .53)
	Last 600	.25 (−.04, .51)	.42** (.14, .64)
SRT2–SRT3	1,000	.15 (−.15, .43)	.41** (.13, .62)
	Last 600	.30* (.01, .55)	.60*** (.37, .76)

* *p* < .05. **p < .01. ***p < .001.

As in Experiment 1, overall response times were highly stable across sessions (probable trials, *r*s = .82–.89; improbable trials, *r*s = .79–.83) but the procedural learning effect showed poor stability between Sessions 1 and 2, as reported in Table 8. Although there was a numerical improvement in stability between Sessions 2 and 3 which was most evident for the regression slope metric, this numerical increase in stability was not statistically significant (overall: *z* = −0.38, *p* = .70; last 600 trials: *z* = −1.08, *p* = .28).

**Figure 6.**
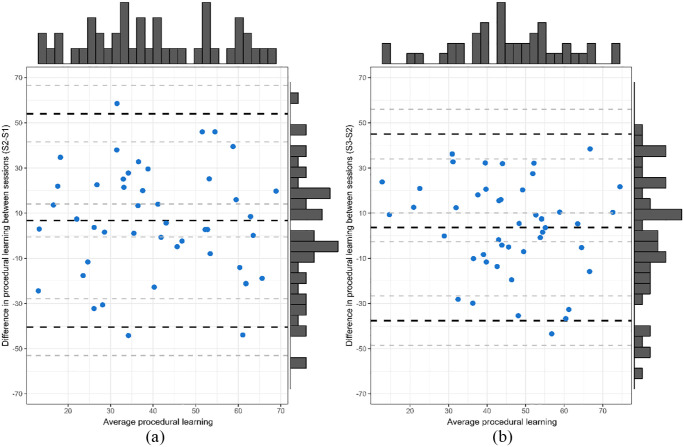
Plot of the mean of the two measurements against the differences between procedural learning in (a) Session 1 and 2 and (b) Sessions 2 and 3.

The Bland–Altman’s 95% limits of agreement range between −40.47 and 54.03 for Sessions 1 and 2 and between −37.62 and 45.03 for Sessions 2 and 3 (Figure 6). Almost all participants fell within the limits of agreement; however, the limits of agreement lacked precision (i.e., the magnitude of the procedural learning effect lacks consistency whereby performance on one session is not necessarily replicated in another possibly reflecting a high degree of measurement), thus revealing poor agreement between measures. Yet, the Bland–Altman plot for Sessions 2 and 3 shows narrower limits of agreement, indicating an improvement in agreement for later sessions.

#### H5–H8: relationship between procedural learning and cognitive measures

The random slopes were used as a measure of procedural learning for analyses of individual differences as this method of calculation demonstrated the highest split-half and test–retest reliability, especially between Sessions 2 and 3 (see Additional Analyses 5 for the Bayes factors and credible intervals for the bivariate correlations between procedural learning and cognitive measures).

Procedural learning was not significantly correlated with nonverbal IQ (Session 1: *r* = −.08, BF_10_ = 0.38; Session 2: *r* = .09, BF_10_ = .39; Session 3 = .22, BF_10_ = .87); thus, nonverbal IQ was not used as a covariate in subsequent analyses.

##### Language and literacy

Vocabulary (*r* = .39) was the only significant language or literacy correlate of procedural learning and only in Session 3, indicating that participants with higher vocabulary skills also demonstrated greater procedural learning. However, this correlation did not survive Holm–Bonferroni correction (see Table 9). Nonetheless, Bayesian correlations revealed that there was evidence against the null hypothesis (BF_10_ = 7.55).

**Table 9.** Correlation matrix between procedural learning and cognitive measures.

Measures	Procedural learning Session 1	Procedural learning Session 2	Procedural learning Session 3	Procedural learning All Sessions
Age	.22 (−.08, .49)	−.04 (−.33, .25)	−.05 (−.34, .24)	.04 (−.25, .33)
Literacy				
Word reading	.04 (−.27, .33)	.014 (−.16, .41)	−.002 (−.30, .30)	.08 (−.21, .36)
Nonword reading	.02 (−.28, .32)	.08 (−.21, .36)	.06 (−.24, .35)	.11 (−.18, .39)
Spelling	.20 (−.11, .47)	.25^†^ (−.05, .50)	.03 (−.27, .32)	.22 (−.08, .48)
Language				
Vocabulary	−.08 (−.37, .22)	.11 (−.18, .39)	.39**^a^ (.11, .61)	.23 (−.06, .48)
Nonword repetition	−.04 (−.33, .26)	−.10 (−.38, .19)	−.24 (−.50, .06)	−.23 (−.48, .06)
Recalling	−.16 (−.43, .15)	−.15 (−.42, .15)	−.29^†^ (−.53, .01)	−.24^†^ (−.50, .05)
Nonverbal IQ				
Matrix reasoning	−.08 (−.37, .24)	.09 (−.21, .38)	.22 (−.08, .49)	.07 (−.23, .36)
Attention				
PVT median	−.28^†^ (−.53, .02)	−.45**^a^ (−.66, −.19)	−.25 (−.51, .05)	−.45**^a^ (−.66, −.19)
PVT reciprocal	.30* (.00, .55)	.49***^a^ (.23, .68)	.25^†^ (−.05, .51)	.49***^a^ (.24, .69)
PVT tau	−.18 (−.46, .13)	−.14 (−.41, .16)	−.19 (−.47, .11)	−.28^†^ (−.53, .01)

† *p* < .10; *p < .05; **p < .01; ***p < .001; bold—Correlations that survived correction for multiple comparisons. PVT: psychomotor vigilance task.

a Correlations with Bayes factor equal or bigger than 3.

##### Attention

A positive and significant correlation was observed between procedural learning and sustained attention for Sessions 1 (median: *r* = −.28; BF_10_ = 1.46, reciprocal: *r* = .30, BF_10_ = 1.90) and 2 (median: −.45, BF_10_ = 29.88; reciprocal: *r* = .49, BF_10_ = 64.40); this association was smaller and nonsignificant for Session 3 (median: *r* = −.25, BF_10_ = 1.11; reciprocal: *r* = .25, BF_10_ = 1.15). As shown in Table 8, there were negative and nonsignificant correlations for the tau parameter, which indexes intraindividual variability (*M* = 63.84, *SD* = 28.73) for all sessions (SRT1: *r* = −.18, BF_10_ = .63; SRT2: *r* = −.14, BF_10_ = .48; SRT 3: *r* = −.19, BF_10_ = .68).

Given the negative relationship between attention and procedural learning, whereby individuals with better attentional skills showed better procedural learning, correlations between tau and SRTT stability were explored to examine whether individuals with high levels of intraindividual variability in attention would also show less stability in the SRTT. Using a medium split approach, the sample was divided into high- and low-tau groups. With respect to Sessions 1 and 2, moderate stability was found for both low-tau (*r* = .29) and high-tau (*r* = .42) groups (the numerical difference was nonsignificant: *z* = −.33, *p* = .74). However, there was a marked difference between low- and high-tau groups for test–retest stability across Sessions 2 and 3, with the low tau group showing higher test–retest stability (*r* = .73) than the high-tau group (*r* = .26). Importantly, the difference between these correlations was statistically significant: *z* = 2.07, *p* = .04. That is, participants with lower intraindividual variability on the measure of sustained attention were also those with more stable procedural learning effects on the SRTT across Sessions 2 and 3.

### Discussion

Experiment 2 examined the stability of procedural learning over three sessions, as well as the relationship between procedural learning and attention and language measures. As in Experiment 1, the procedural learning effect was robust in all sessions. While there was some evidence of a numerical increase in reliability for the later sessions for both split-half and test–retest reliability, these improvements were not statistically significant, and stability remained suboptimal. Procedural learning positively and significantly correlated with sustained attention and, to a lesser extent, vocabulary, with the latter not surviving correction for multiple comparisons.

As predicted, the test–retest reliability of the SRTT showed numerical (but not statistical) improvements across sessions, with stability slightly higher for later sessions. Indeed, the highest level of stability in the current experiment was between Sessions 2 and 3 when using random slopes as the index of learning, *r* = .60 (.37, .76). This is more akin to the stability reported by Siegelman and Frost (2015; *r*(76) = .47) and West et al. (2021; *r*(46) = .70), although in these studies this level of stability was found across two sessions rather than three. Overall, the highest stability was observed when focusing on the procedural learning effect on the last three epochs, which aligns with Conway and colleagues’ (2019) suggestion that the inclusion of earlier stages of procedural learning, when learning is not yet robust, may reduce test–retest reliability. Nonetheless, the linear mixed effects model and the Bland–Altman plots indicate that, even though increasing the number of sessions reduced practice effects, there was still a significant procedural learning improvement between Sessions 2 and 3. This may indicate that additional sessions may be required to reach a plateau in procedural learning; while this would be theoretically important to ascertain, it would limit the practical utility of using the SRTT in clinical or developmental research. Furthermore, it is unclear whether the superior reliability for later sessions results from participants having more training opportunities or more consolidation opportunities. Future research would be needed to examine what underlies the better stability across time. This pattern was observed despite adopting distinct, though similar, sequences at each session, with the aim of reducing practice effects (Palmer et al., 2018). In a recent meta-analysis on retest effects in working memory tasks, improvements in performance were observed until the 7th session, yet they were no longer significant after the 4th administration (Scharfen et al., 2018). Trial variability (i.e., the variance in the response times for probable and improbable trials) also decreased across sessions, further suggesting that measurement error decreased across sessions, with an increase in the signal-to-noise ratio (Chen et al., 2021; Rouder & Haaf, 2019). Nevertheless, it should be emphasised that the increase in stability over sessions observed here was not statistically significant. Finally, with the present sample size of approximately 50 participants, we cannot be completely confident in the point estimates (as suggested by the sensitivity analyses conducted in Supplementary Materials 1). Thus, this effect warrants replication in future work.

Contrary to our hypotheses, there was minimal evidence of an association between procedural learning and language. We found only a moderate correlation that did not survive correction for multiple comparisons, between procedural learning and vocabulary Session 3. It is worth noting that this aspect of language is proposed to be more highly associated with declarative than procedural memory (Ullman, 2004). Notably, and also counter to Ullman (2004), there were no associations between procedural learning and measures of grammar, phonology, and decoding. As with previous studies that have failed to find robust associations, it may be that the suboptimal test–retest reliability of the SRTT results in an underestimation of the true effect size (Rouder et al., 2019).

The most robust association in the present experiment was between attention and procedural learning, particularly in Sessions 1 and 2. This finding is consistent with the results obtained by Sengottuvel and Rao (2013) and West et al. (2021), and points to attentional resources playing a facilitatory effect in the magnitude and stability of procedural learning on the SRTT as individuals with lower intraindividual variability (as indexed by tau) showed better stability, particularly for later sessions. The decrease in the magnitude of the correlation between attention and procedural learning in Session 3 may be related to the findings obtained by Thomas and colleagues (2004), which demonstrated that a decrease in parietal activity, a brain region which plays a role in visual attention and spatial orienting, occurred once the sequence became more predictable. Thus, tentatively, the smaller correlation in Session 3 may indicate that as the sequence became more predictable with increasing practice, this worked to reduce reliance on attentional resources (Thomas et al., 2004). However, it remains for future research to test this hypothesis directly.

## General discussion

Procedural learning is thought to be a fundamental component of the memory system, crucial for encoding, storing, and retrieving rule-governed knowledge that underlies motor and cognitive abilities (Cohen & Squire, 1980). Research into this vital memory system is often reliant on the SRTT; however, questions have been raised about the reliability of this task. Here, we present a systematic examination of the reliability of procedural learning as measured by the SRTT, with the important aim of identifying extrinsic design features (i.e., similarity of sequences learned over sessions, number of sessions, stimulus presentation rate) and participant characteristics (i.e., attention, age, see Supplementary Materials 4) that could influence reliability. In Experiment 1, manipulation of the levels of similarity between sequences learned at Sessions 1 and 2 revealed a positive relationship between similarity and the procedural learning effect, yet the participant-level stability of the effect was low irrespective of similarity. A follow-up to this found that despite further manipulations of sample (age) and task (ISI) characteristics (see Supplementary Materials 4) the test–retest reliability of the SRT remained low. Experiment 2 examined the effect of training over three sessions. However, irrespective of experimental manipulations and participant characteristics, the test–retest reliability of the SRTT remained persistently suboptimal (*r* < .70). When all participants who performed the SRTT without an ISI (*N* = 184) were included in the analyses to obtain an overall estimate of reliability across experiments, the test–retest reliability was still well below acceptable standards, random slopes, 600 trials: *r* = .33 (.19, .45); see more details in Supplementary Materials 1.

Importantly, the issue of reliability of procedural learning tasks is not specific to the SRTT, as other measures of procedural memory have also been found to show poor reliability (e.g., artificial grammar learning: Kalra et al., 2019; probabilistic classification task: Kalra et al., 2019; Hebb task: West et al., 2018; auditory and visual statistical learning tasks: Arnon, 2020). Weak correlations among different tasks thought to index procedural memory (Arnon, 2020; Kalra et al., 2019; Siegelman & Frost, 2015; West et al., 2018) have led researchers to question unitary accounts of procedural memory, in support of more componential views (Arciuli, 2017). Yet, it is unlikely that correlations between these measures would emerge, even if they capture the same underlying construct given that the degree of attenuation is impacted by the poor reliability of both measures (Spearman, 1910). Beyond this, the issues with reliability are not specific to procedural memory, with similar findings reported for other classic, widely used experimental paradigms in cognitive psychology (e.g., Stroop task, Flanker task: Haines et al., 2020; Hedge et al., 2018; von Bastian et al., 2020). This phenomenon is referred to as the “reliability paradox” (Hedge et al., 2018), where experimental paradigms known for eliciting robust effects fail to capture stable individual differences. The reliability paradox is thought to be a consequence of the use of experimental tasks in individual differences research which have been designed to reduce variability between individuals to ensure that the phenomenon of interest is captured. Unfortunately, this reduction in between-subject variability has consequences for individual differences as it limits the ability of a test to differentiate between individuals (Hedge et al., 2018).

The use of difference scores has been suggested as a contributing factor to poor reliability as such scores can reduce the signal-to-noise ratio (Hedge et al., 2018). Despite the debates surrounding the limitations of adopting difference scores as indices of the construct of interest (Hedge et al., 2018), differences scores were used in this experiment to estimate split-half reliability often produced within-session stability estimates between .50 and .93, with the exception of the third session of Experiment 2, thus, revealing mostly adequate internal consistency in participants’ performance between halves (odd-numbered and even-numbered trials). Furthermore, the use of random slopes as an index of procedural learning did not significantly improve reliability. Importantly, this suggests that one should not dismiss difference scores as being intrinsically unreliable. This also raises a clear distinction between within-session and across-session stability in the SRTT. Higher within- than across-session stability of the SRTT has been found in previous studies of children and adults (e.g., West et al., 2018, 2021), with this pattern mirrored in studies using other measures of sequential learning (Hebb task—e.g., Bogaerts et al., 2018; West et al., 2018; statistical learning e.g., Arnon, 2019—although this pattern was only found for a visual version of the task and not for linguistic/nonlinguistic versions). One simple explanation for why we observe higher within-session than across-session reliability could be due to temporal differences, such that there is a decrease in the magnitude of correlations between trials as the number of intervening trials increases (Wagenmakers et al., 2004). More specifically, while short-scale fluctuations are present when computing split-half reliability where even–odd trials are compared, more distant points are compared for the test–retest reliability which, in the present studies, occurred 1 week apart.

However, this explanation does not account for why we do not see the same disparity between within- and across-session stability for declarative tasks (Buchner & Wippich, 2000; LeBel & Paunonen, 2011; Ward et al., 2013). Kalra et al. (2019) and West et al. (2018) observed that the test–retest reliability of all procedural learning measures was inferior to those of declarative measures. In West et al. (2018), for example, test–retest reliability for the nonverbal immediate serial recall and dot locations tasks test–retest .71 and .57 and split-half reliability was .68 and .76, respectively. This is perhaps in part due to the complex nature of procedural learning itself and the multifaceted nature of the tasks used to measure this poorly defined construct (Bogaerts et al., 2021). Addressing this issue is made even more complex by the interchangeable use of tasks (e.g., Artificial Grammar Learning, Weather Prediction task) that are claimed to tap into procedural memory as a unified ability, despite their computational and modality differences.

Recently, it has been argued that poor test–retest reliability of some tasks (e.g., Stroop task, Flanker test), well known for producing robust effects at the group level, may be related to the methods adopted to analyse their psychometric properties. Haines and colleagues (2020) show adequate test–retest reliability when using Bayesian hierarchical modelling which more closely captures individuals’ performance and accounts for within-subject variability, but suboptimal test–retest reliability when using difference scores. In these models, instead of ignoring uncertainty, as is the case when using point estimates (e.g., mean), which may underestimate test–retest reliability, hierarchical Bayesian models aim to closely represent the data generating process. By using generative modelling, a single model is able to integrate information at the individual and group levels when estimating parameters, accounting for our assumptions and hypotheses from the trial-by-trial response times at the individual level to the overall distribution of individual differences across people (see Haines et al., 2020). Yet here we aimed to explore the impact of experimental manipulations on reliability using statistical methods/measures comparable to previous research (i.e., by estimating the procedural learning effect separately for each session). Future studies may aim to apply the methods applied by Haines et al. (2020) to the SRTT to determine whether it would better capture the stability of the procedural learning effect across sessions.

Previous studies have noted an association between attention and procedural learning (Arciuli, 2017; Sengottuvel & Rao, 2013; Shanks & St. John, 1994; West et al., 2021); however, here, we carried out the first investigation of whether attention influences the stability of procedural learning. Exploratory analyses in Experiment 2 and the Supplementary Experiment (see Supplementary Materials 4) suggest that participants with better attention skills (lower tau) showed more stable procedural learning across sessions than those with worse attention. Thus, these results may lend support to the hypothesis that fluctuations in attention during the task could lead to lower test–retest reliability. One interesting prediction that arises here is that fluctuations of attention may exert lower impact on split-half reliability as this type of stability would be captured by both halves of the task due to the time proximity between even and odd trials. This warrants a systematic assessment of the attention skills during the SRTT using online measures of attention such as pupillometry to better determine its relationship with procedural learning both within and across sessions. A second interesting prediction here is that if attentional skills influence the stability of procedural learning on the SRTT task, then children would be expected to show poorer test–retest reliability than adults as their attentional skills are under development (Levy, 1980). Indeed, this pattern of lower retest reliability has been observed in children by West et al. (2018, 2021), despite somewhat comparable split-half reliability to adults, children: West et al., 2018—SRT1, *r* = .75; SRT2, *r* = .49 (500 trials); West et al., 2021—SRT1, *r* = .51; SRT2, *r* = .62 (1,000 trials); adults: West et al., 2021—SRT1, *r* = .84; SRT2, *r* = .92 (1,000 trials).

Fluctuations in procedural learning over time may also be related to changes in performance between measurement points due to individual differences in consolidation and other learning-related strategies adopted at test and retest. This could also account for the higher within- than across-session stability. In line with this, Scharfen et al. (2018), in a recent meta-analysis observed that participants reached a plateau later in working memory tasks compared with other cognitive ability tests. Authors argued that more complex tasks lead to larger retest effects because more test-specific strategies can be developed compared with easier tasks for which strategies do not apply. In the SRTT, this may be accompanied by, or occur due to the development of explicit awareness, as suggested by Stark-Inbar et al. (2017). Thus, the numerically higher test–retest reliability for later sessions observed in Experiment 2 would be expected given that participants’ may be reaching a plateau in their learning effect—seen as a reduction in the practice effects for later sessions. In addition, the strategies adopted for later sessions would potentially be more similar as most participants would already possess some awareness of the presence of an underlying sequence. Future research may aim to explore the trajectory of learning on the SRTT across sessions until no practice effects are observed and its impact on reliability. Alternatively, participants could be asked to perform the SRTT in an initial practice session until each reaches a plateau in performance, only then reliability would be assessed in two separate sessions. However, as a first step, due to the small sample size of Experiment 2 and our sensitivity analysis suggesting that a sample size of at least 100 participants is necessary to obtain a more precise estimate of the test–retest reliability, future work is required to determine whether the superior reliability in later sessions emerges under similar experimental conditions.

It is important to consider the extent to which poor across-session reliability of procedural learning on the SRTT may impact our ability to adequately test the predictions of models of language and literacy acquisition, namely the declarative/procedural model (Ullman, 2004). This model predicts that the procedural memory system is involved in the development of language and literacy abilities that underlie aspects of rule-based learning. Yet, given that procedural learning tasks may fail to capture participants’ true procedural learning abilities, attenuation of the correlation between the constructs of interest is likely to occur. Thus, unsurprisingly, Experiment 2 provided no support for the declarative/procedural model (Ullman, 2004). While there was a weak positive correlation between procedural learning and vocabulary (which would not necessarily be a firm prediction of the declarative/procedural model), there were no other significant correlations with other language/literacy measures that have been claimed by this model to be associated with procedural learning (i.e., grammar, phonological skills). Nevertheless, a positive relationship between procedural learning and attention *was* observed in Experiment 2 (and also in the experiment presented in Supplementary Materials 4), irrespective of the reliability issues and possible attenuation of correlations between measures. Thus, it is also possible that this result reflects a genuine lack of support for the declarative/procedural model (Ullman, 2004) and/or poor measurement of procedural learning (Enkavi et al., 2019).

Finally, individual differences research assumes that there are stable differences between individuals in the construct of interest which may influence individuals’ accumulated experience/learning over the long term, which, if adequately captured, would likely result in adequate stability. However, it is possible that the poor reliability of the procedural learning effect does not reflect a problem with the paradigm. Instead, this may indicate that there is insufficient variability in the procedural learning effect, as it may be sufficient for a minimum level of procedural learning ability to facilitate acquisition of cognitive and motor skills and habits. Therefore, the magnitude of the difference scores may carry only limited meaning, instead it may be more important whether the individual is able to extract any knowledge from the task, irrespective of its magnitude. This is in line with Reber’s (1989) proposal that procedural learning due to being evolutionarily old differs substantially from declarative memory as it is expected to show little between subject-variability. Following from this, if individuals do not differ enough from one another, then measurement fluctuations will lead to substantial changes in ranking order.

While the various experimental attempts to improve the test–retest reliability of the SRTT were not effective here, there are other potential manipulations to explore. For instance, a critical design element of SRTTs is the number of trials. We carried out a preliminary exploration of this factor with simulation work presented in Additional Analyses 1 and demonstrated that the ratio of probable to improbable trials can influence test–retest reliability. While researchers have considered the number of trials in the SRTT (e.g., West et al., 2021), the focus tends to be on the overall number of trials, rather than the number of trials per condition as recommended by Rouder et al. (2019). Further experimental work is necessary to determine whether increasing the number of trials in the improbable condition could reduce measurement error, while considering the potential consequences for the size of procedural learning effect. Furthermore, considering the findings by West et al. (2021), which suggest that attention during the SRTT, but not procedural learning, predict children’s reading, grammatical, or arithmetic skills, it is crucial to determine if attention mediates the relationship between procedural learning and language/literacy measures or whether poorer attentional skills represent an additional risk factor for procedural learning deficits in children/adults with Dyslexia.

Finally, Bayesian hierarchical models have been shown to be useful in estimating the degree of attenuation in correlations between measures (e.g., attentional control; Rouder & Haaf, 2019; von Bastian et al., 2020), with trial noise and true variability being estimated separately (Rouder & Haaf, 2019). Future research would benefit from exploring the use of these approaches for procedural learning. Regardless of the consistent suboptimal test–retest reliability of the procedural learning effect, the SRTT has reliably produced robust evidence of learning across populations and settings. Thus, while the current set of experiments challenges its suitability for individual differences research (Enkavi et al., 2019), there is little doubt that the SRTT is still a valuable paradigm for group-level experiments.

In conclusion, the probabilistic SRTT used here produced robust procedural learning effects across three experiments, irrespective of samples and testing conditions. Yet, despite some weak evidence of improvement in stability due to the experimental manipulations presented here, it remains suboptimal. Future research should focus on understanding (1) the discrepancy between within- and across-session reliability (e.g., temporal dynamics, consolidation processes) and (2) whether there are more sensitive analytical methods that can be used to assess across-session reliability (e.g., Haines et al., 2020). It will also be important to further investigate the potential role of attention in procedural learning, particularly in individuals vulnerable to poor attention (e.g., including those with dyslexia/DLD). Thus, until these questions are answered, it is not possible to use the SRTT to test the boundaries of the Procedural/Declarative model.

## Supplemental Material

sj-pdf-1-qjp-10.1177_17470218241232347 – Supplemental material for Reliability of the serial reaction time task: If at first you don’t succeed, try, try, try again Supplemental material, sj-pdf-1-qjp-10.1177_17470218241232347 for Reliability of the serial reaction time task: If at first you don’t succeed, try, try, try again by Cátia M Oliveira, Marianna E Hayiou-Thomas and Lisa M Henderson in Quarterly Journal of Experimental Psychology

## Notes

1. Available at https://osf.io/fn9mw/.

2. Ratio scores were also computed taking individual differences in baseline reaction time (RT) into account by dividing participants’ difference scores by their overall mean RT per session (Ratio1; Kalra et al., 2019; West et al., 2021) or by their improbable RTs (Ratio 2; Urry et al., 2018). These yielded lower reliability than the regression slope scores; full details are reported in Additional Analyses 8.
