# Supplementary material for: Reliability of the serial reaction time task: If at first you don’t succeed, try, try, try again
Source: Q J Exp Psychol (Hove). 2024 Mar 7;77(11):2256–82. doi: 10.1177/17470218241232347 (PMC11529135; doi:10.1177/17470218241232347)
Supplement: sj-pdf-1-qjp-10.1177_17470218241232347 – Supplemental material for Reliability of the serial reaction time task: If at first you don’t succeed, try, try, try again [file sj-pdf-1-qjp-10.1177_17470218241232347.pdf]

SUPPLEMENTARY MATERIALS

1. Sample size 2
2. Explicit Awareness 5
   - Generation Tasks 5
   - Results 6
     - Experiment 1 6
     - Experiment 2 7
3. Experiment 2: Cognitive Measures 8
4. Supplementary Experiment 9
   - Abstract 9
   - Introduction 9
   - Methods 10
     - Participants 11
     - Procedure 11
     - Statistical analyses 12
   - Results 13
     - H1: Procedural learning in the SRT task - Effect of ISI 13
     - H4: Procedural learning in the SRT task - Age effects 17
     - H2 and H3: Reliability 19
     - H6: Explicit awareness 23
     - H5 and H6: Relationship between procedural learning and attention, and explicit awareness 24
   - Discussion 25

References 27

1. Sample size

To investigate the effect of sample size on the test-retest reliability, we adopted the same approach as Farkas et al. (2023). All participants who completed the SRTT without an ISI (N = 184) were included in the analysis as we found an effect of ISI membership on test-retest reliability (more details are available in section 4 which describes in detail the supplementary experiment). To account for potential differences between experiments, *Experiment* was entered as a predictor in the linear mixed model alongside *Probability*.

Figure 1

Mean and 95% CI response times for probable and improbable trials per Epoch and Session (Session 1 on the left, Session 2 on the right) for each experiment.

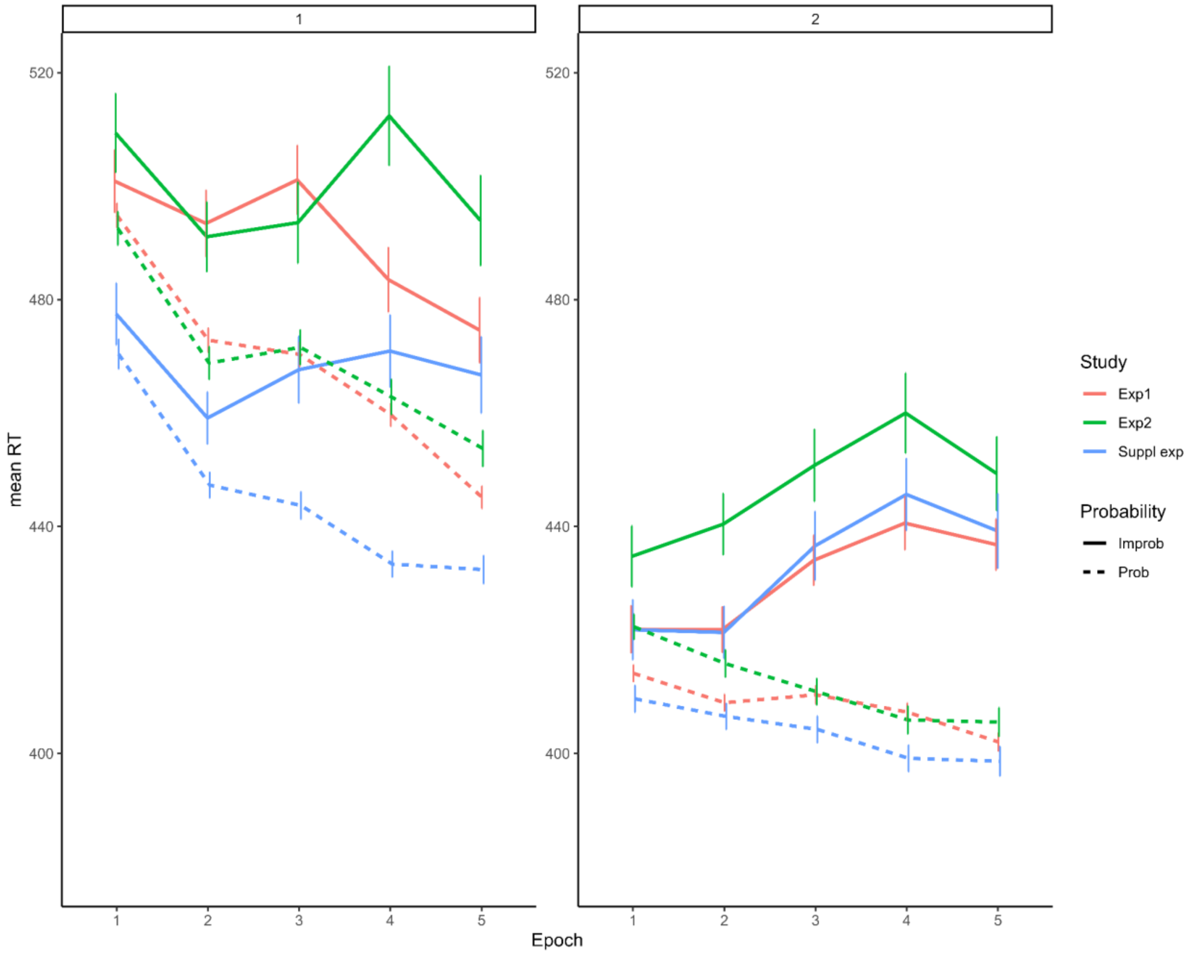

In the permutation analyses, the number of participants was randomly sampled in steps of ten from 10 to 180 participants. This process was repeated 100 times per sample size and the average reliability, and its confidence interval were computed across these iterations. As observed by Farkas

et al. (2023), increasing the sample size resulted in a more precise estimation of the test-retest reliability, both for difference scores and random slopes. Furthermore, similarity to their findings, although less accentuated, sample sizes above 100 participants contributed to only limited gains in precision. Further details on the test-retest reliability for all participants who performed the no-ISI version of the SRTT are included in Table 1, as before, there was a small improvement in the reliability estimate for model-based estimates than difference scores, however, both measures are thought to be capturing the same construct as suggested by the high correlations between these measures for both sessions (Session 1: .86; Session 2: .90)

Figure 2

Permutation analyses of the effect of sample size on the reliability of the procedural learning effect (top row) and its confidence intervals (bottom row) for difference scores (A) and random slopes (B).

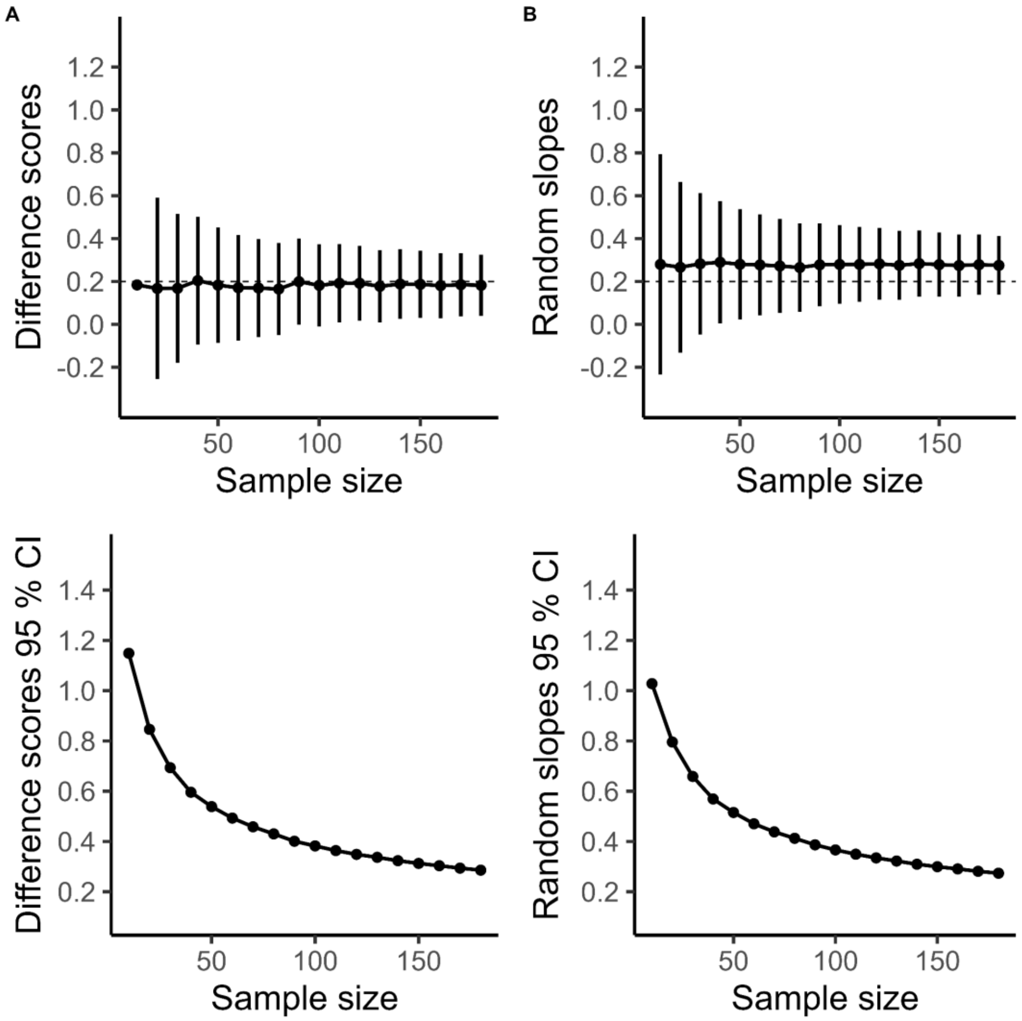

Table 1

Pairwise test-retest reliability of the procedural learning measures

TRIALS	Test-retest reliability			
	Difference scores	Ratio1	Ratio2	Random slopes
Overall	.18* (.04, .32)	.21** (.06, .35)	.21** (.06, .35)	.28*** (.14, .41)
Last 600 trials	.17* (.01, .30)	.21** (.06, .34)	.24** (.10, .37)	.33*** (.19, .45)

Note. *p < .05, **p < .01, ***p < .001; Ratio1 was computed taking individual differences in baseline RT into account by dividing participants' difference scores by their overall mean RT per session (Improbable trials - Probable trials)/(Improbable + Probable)/2); Ratio 2 was computed taking individual differences in baseline RT into account by dividing participants' difference scores by their improbable RT per session (Improbable trials - Probable trials)/Improbable trials

2. Explicit Awareness

Generation Tasks

To measure explicit sequence memory, after completion of the SRTT, participants completed a generation task (Wilkinson & Shanks, 2004). This task presented the same four black outlined rectangles as in the main SRTT. Following Wilkinson and Shanks (2004), participants were instructed as follows: first they were asked if they had noticed a pattern in the task *“Did you notice a pattern in the task? Press: Y for YES, N for NO, or U for UNSURE”*; and then informed that a sequence was embedded in the task with participants being asked to “guess” what the pattern was by making 100 key presses using the following instructions adapted from Wilkinson and Shanks (2004):

Inclusion instructions

During the reaction time trials, you may have noticed that the smiley face appeared in a regular repeating sequence. (Do not worry if you did not notice this; the sequence was designed to be very difficult to detect). Now in the final stage of the experiment we will see how much (if anything) you have learned about the sequence. You will have to do a slightly different task in this final block of trials. Your reaction time will no longer be measured. Instead of responding to the position of the smiley face, what we would like you to do is to press the keys 100 times, attempting to freely generate the sequence that you saw in the reaction time phase. Each time you press a key, the smiley face will appear in the appropriate box. It will remain on the screen until you press a further key. Do not worry if your memory of the sequence is poor; just try to generate the sequence as best as you can. Please avoid pressing the same key on successive occasions. The computer will tell you when you have made 100 keypresses. Try to be as accurate as possible.

Press space to start.

In the supplementary experiment, after completing the task previously described, participants were additionally asked to complete an additional task - exclusion condition, in which participants were instructed to avoid generating the sequence acquired during the SRTT. The following instructions were used following the instructions also adapted from Wilkinson and Shanks (Wilkinson & Shanks, 2004):

Exclusion Instructions

In the final stage of the experiment we will use a different method to see how much (if anything) you have learned about the sequence. As in the last block you must press the keys 100 times but this time attempting to freely generate a sequence that is as DIFFERENT as possible from the one you saw in the reaction time phase. If you can remember particular parts of the sequence, then you should avoid generating them. Each time you press a key, the smiley face will appear in the appropriate box. It will remain on the screen until you press a further key. Don't worry if your memory of the sequence is poor; just try as best you can to avoid generating the sequence. Please avoid pressing the same key on successive occasions. The computer will tell you when you have made 100 keypresses.

Press space to start

Both in the inclusion and exclusion conditions, the levels of explicit awareness were analysed using the following measures: the number of total and distinct triplets and the longest consecutive string of elements of the sequence. To determine whether participants presented explicit awareness beyond chance levels, chance performance was determined by randomly generating 16000 sequences of 100 keypresses which was compared to participants' scores by conducting one sample t-tests as previously done by Lee & Tomblin (2015). Chance level performance was estimated by randomly generating 16000 sequences of 100 keypresses with the average chance level of triplets being estimated at 29.96, distinct triplets at 10.4, and of longest sequence elements at 5.84.

Results

Experiment 1

40/98 participants indicated noticing a pattern (with 37 participants responding negatively, and 21 unsure).

In the generation task, participants recalled on average 9.31 distinct triplets (SD = 1.96) and 6.48 elements of the 12-item sequence (SD = 1.70). One sample t-tests revealed significant differences between the chance level and participants' performance (total triplets: $t(96) = 7.76$, df = 96, $p < .001$; distinct triplets: $t(96) = -5.48$, $p < .001$; elements of the sequence: $t(96) = 3.73$, $p < .001$), with participants generating significantly less distinct triplets but more total triplets and elements of the sequence than chance.

Pearson correlations between regression slopes for both sessions and performance on the generation task were negligible and non-significant ($ps < .05$) for the total number of triplets generated

(session1: r = -.08, p = .452, BF_{10} = .27; session2: r = -.06, p = .566, BF_{10} = .50), different triplets (session1: r = -.04, p = .674, BF_{10} = .29; session2: r = .06, p = .594, BF_{10} = .47) and longest sequence (session 1: r = .04, p = .685, BF_{10} = .43; session2: r = -.01, p = .932, BF_{10} = .25). Similarly, a negligible relationship between similarity and explicit awareness was observed when correlating both measures of explicit awareness with similarity levels, for total triplets (r = .06, p = .57, BF_{10} = .24), different triplets (r = -.003, p = .975, BF_{10} = .30) and longest sequence recalled (r = -.06, p = .541, BF_{10} = .24). This indicates that the levels of explicit awareness are not associated with procedural learning nor with similarity.

As in the present experiment, explicit awareness has been frequently observed in the SRTT after extended training (Shanks et al., 2005; Wilkinson & Shanks, 2004). This has been found to occur despite attempts to reduce explicit awareness by adopting a probabilistic SRTT (Jimenez & Mendez, 1999) and the absence of an interstimulus interval (Destrebecqz & Cleeremans, 2001). Yet, explicit awareness was not associated with the amount of sequence learning in our experiment. It is possible that the low reliability of the SRTT could lead to the underestimation of the true relationship between sequence and explicit learning (Arnon, 2019). However, a substantive interpretation of these results is supported by the findings of Song et al. (2007), who showed that even when participants were exposed to the sequence before performing the SRTT, this did not improve sequence learning. Taken together, this pattern of results strongly suggests that implicit learning develops continuously with its time course being unaffected by explicit knowledge.

Experiment 2

After completion of the SRTT in the second session, participants were asked to answer two questions related to explicit awareness and complete a free generation task. The first question was related to whether participants noticed a pattern in the SRTT, to which 22 participants responded affirmatively, 13 negatively, and the remaining 11 were unsure about the presence of a sequence. The second question referred to the timing of awareness of the presence of a pattern, 13 participants indicated that they noticed the sequence in the first session, 12 in session 2 and 7 in session 3. The remaining participants (N = 14) indicated that they never noticed a pattern. In the generation task, participants recalled on average 9.35 distinct triplets (SD = 1.39; max = 12) and 6.46 elements of the 12-item sequence (SD = 1.44; max = 12). One sample t-test against chance levels (distinct triplets: M = 10.40; elements of the sequence: M = 5.84) revealed a similar pattern to experiment 1 with participants generating significantly fewer distinct triplets ($t(45) = -5.15$, $p < .001$) and more elements of the sequence ($t(45) = -2.90$, $p = .006$) than chance levels.

Unlike experiment 1, there was evidence of a small negative, non-significant ($ps < .05$) association between procedural learning and the level of explicit awareness, both when this was indexed by the number of total triplets recalled (session 1: $r = -.005$, $BF_{10} = .34$, session 2: $r = -.06$, $BF_{10} = .36$; session 3: $r = .06$, $BF_{10} = .36$), different triplets (session 1: $r = -.15$, $BF_{10} = 0.52$, session 2: $r = -.08$, $BF_{10} = .57$; session 3: $r = -.18$, $BF_{10} = .64$), and by the longest sequence recalled by the participants (session 1: $r = -.14$, $BF_{10} = .49$, session 2: $r = -.08$, $BF_{10} = .37$, session 3: $r = -.007$, $BF_{10} = .33$).

3. Experiment 2: Cognitive Measures

Table 2

Mean (and SD) scores for all background measures

	Test	Raw scores			Standardised scores	
		Mean (SD)	Range	Max	Mean (SD)	Range
**Age**	-	20.09 (2.09)	17 - 34			
**Matrix reasoning**	WASI – II	21.71 (2.57)	14 - 26	30	52.04 (7.13)	32 - 68
**Word reading**	WIAT – III	67.78 (3.32)	59 - 73	75	97.75 (7.22)	81 - 115
**Nonword reading**	WIAT – III	42.54 (4.62)	32 - 49	52	98.11 (11.53)	72 - 114
**Spelling**	WIAT – III	55.51 (3.98)	47 - 63	63	109 (11.21)	78 - 134
**Sentence Recall**	CELF 5	69.92 (4.83)	59 - 76	26	11.26 (2.38)	7 - 15
**Nonword Repetition**	CToPP 2	20.53 (2.33)	16 - 26	30	55.28 (25.15)	9 - 98
**Vocabulary**	WASI – II	36.28 (3.24)	30 - 43	59	NA[1]	NA[1]

Note. CELF - 5 UK, Clinical Evaluation of Language Fundamentals - Fifth Edition (Wiig et al., 2013); CToPP-2, Comprehensive Test of Phonological Processing 2 (Wagner et al., 2013); WASI - II, Wechsler Abbreviated Scales of Intelligence – Second edition (Wechsler, 2011); WIAT – III, Wechsler Individual Achievement Test - Third UK Edition (Wechsler, 2009); [1] Raw scores were used in all analyses.

4. Supplementary Experiment

Abstract

The Serial Reaction Time task (SRTT) is a robust measure of procedural learning despite consistent poor stability across sessions (rs < .70), except for West et al. (2021). In this experiment, we aimed to more closely replicate their design by adopting an interstimulus interval (ISI) of 250 ms and recruiting participants from a wider age range (18-60 years). Counter to our predictions, the test-retest reliability was poor for both groups with and without an ISI (rs < .50), suggesting that other design features may have contributed to the superior stability observed by West et al. (2021).

Introduction

Whilst manipulating the similarity between sequences in Experiment 1 and increasing the number of sessions in Experiment 2 contributed to a larger experimental effect for later sessions and a decrease in trial variability, the test-retest reliability of the SRTT remained suboptimal. This may be associated with the small variability between participants when compared to within-subject variance in the procedural learning effect, which diminishes the test's capacity to effectively differentiate participants' performance (Hedge et al., 2018; Spearman, 1910). In West et al. (West et al., 2021), where substantially higher test-rest reliability was observed, both the magnitude of the procedural learning effect and the variability across participants were considerably higher than our previous experiments. Thus, in this experiment we aimed to more closely replicate West and colleagues' (2021), experiment in adults and examine whether participants' wider age range and the presence of an ISI contributed to the higher test-retest reliability.

In this experiment we compared SRTT performance and reliability in two participant groups using the same sequences as in experiment 2: for one group we included an ISI of 250 ms, as in West et al. (West et al., 2021), and for the other there was no ISI, as in Experiment 2. To explore the effect of age on the stability of procedural learning, we recruited participants aged 18-60 years as in West et al. (West et al., 2021), with the groups age-matched. Importantly, this experiment was also run online, allowing us to assess the reliability of an online SRTT. Finally, comparing the relationship between procedural learning and attention in the two ISI groups also offered a further window into the role of attention in the SRTT.

Previous evidence by Destrebecqz and Cleeremans (2001, 2003) has suggested that individuals trained with a 250 ISI develop stronger representations of the underlying sequences, which may

explain the larger procedural learning effects observed by West and colleagues' (2021). If superior procedural learning (and potential automatisation) leads to earlier independence from attention, one would expect that the ISI group would show a smaller correlation between procedural learning and attention (Seger & Spiering, 2011). Alternatively, given the increased duration of the ISI version, it is possible that individual differences in attention may be related to procedural learning in this longer (potentially less engaging) version (Franklin et al., 2016). Thus, these (speculative) explanations were also explored.

The hypotheses (pre-registered at https://osf.io/t78kf) were as follows:

- H1: Participants are expected to demonstrate evidence of procedural learning in both sessions;
- H2: Both groups (with ISI and no-ISI) will show higher split-half reliability than test-retest reliability;
- H3: Higher test-retest reliability will be expected for the ISI group which replicates (West et al., 2021) design more closely than the no-ISI group. It was also predicted that the ISI group will show faster RTs and larger procedural learning effects, and subsequently less evidence of practice effects than the group with no-ISI;
- H4: Slower RTs are expected for older participants, despite similar levels of procedural learning for younger and older participants. Older participants will also be expected to show more stable procedural learning than younger participants;[1]
- H5: Participants in the ISI group will demonstrate higher evidence of explicit awareness by recalling more elements of the sequence, with this difference being more noticeable in the exclusion condition;
- H6: Both ISI groups will demonstrate a positive correlation between procedural learning and sustained attention, as measured by the PVT task; however if the attentional demands of the SRTT diminish once the sequence becomes more predictable, it would be expected that the ISI group would show a smaller correlation between procedural learning and attention than the group with no-ISI in Session 2.

Methods

[1] The effect of age on reliability was explored in Additional Analyses 7

Participants

One hundred and thirty-five participants aged 18-60 years (M = 29.74, SD = 10.01) took part. The sample included monolingual, bilingual and multilingual individuals from 20 distinct nationalities. All participants were proficient English or Portuguese speakers with normal or corrected-to-normal vision. Recruitment was conducted on social media (e.g., Twitter, Reddit and Facebook) and each participant was randomly assigned to the ISI or no-ISI group. The experiment was approved by the Ethics Committee of the Psychology Department at the University of York and each participant gave written informed consent. A power analysis based on the lower bound of the test-retest reliability obtained by West et al. (West et al., 2021) indicated that 35 participants per group would be required to achieve 80% power.

Measures[2]

Serial Reaction Time task. The SRTT used in Experiment 2 was used here. For the group with ISI, there was an interval between trials of 250 ms, whilst for the no-ISI group the following trial started as soon as the response was made. RT and accuracy were measured for each trial. Participants had to respond to each trial to initiate the next trial, regardless of accuracy.

Psychomotor Vigilance task. Sustained attention was measured using a shorter (5 minutes) and online version of the Psychomotor Vigilance task (Reifman et al., 2018) used in experiment 2.

Free generation tasks. Explicit awareness of the training sequence was assessed through two free generation tasks (Wilkinson & Shanks, 2004). After completing the SRTT, participants were informed of the presence of a pattern in the stimuli and were then presented with the outline of four rectangles as in the SRTT and asked to press the same keys to elicit the appearance of the stimuli. The target remained in the corresponding position to the key pressed until a new response was made. In the inclusion task, each participant was asked to generate 100 guesses of the underlying sequence learned in the SRTT; whilst in the exclusion task participants were asked to produce a sequence as different as possible from the training sequence. Each sequence generated by the participants was coded into the number of different triplets recalled. Further details are presented in Supplementary Materials 1.

[2] Online versions of the SRTT, PVT and generation tasks are available at https://gitlab.pavlovia.org/memory-group

Procedure

The experiment used a mixed-subjects design with each participant randomly assigned to an ISI group and performing the SRTT at two time points each separated by roughly one week (M = 7.85, SD = 2.21, range = [5 - 20]). The two underlying sequences of the SRTT were counterbalanced to avoid order effects. Participants were randomly allocated to one of two groups: (i) no interstimulus interval (no-ISI group) or (ii) 250 ms (ISI group). Groups were age-matched to avoid age-related differences in procedural learning. After completion of the SRTT, sustained attention was measured using an adapted version of the Psychomotor Vigilance task (Reifman et al., 2018) to explore the relationship between attention and procedural learning. Additionally, after completion of the SRTT in Session 2 participants performed two free generation tasks aiming to capture explicit knowledge of the sequence. All tasks were programmed using Psychopy 3 (Peirce et al., 2019) and the sessions were run online on the Pavlovia Platform (Bridges et al., 2020).

Statistical analyses

H1 and H4: Mixed effects model. The same procedures as in experiments 1 and 2 were adopted for data treatment and analyses. Two participants were removed from the analyses for both sessions for the ISI group and four participants for the no ISI group, with two of them removed for both sessions and the remaining one from session 1 and the other from session 2.

Given the transition into online testing, a model contrasting online and in-lab results was computed to compare the performance in the SRTT across settings. This model showed similar performance in both settings (in-lab vs online), thus showing that the paradigm is robust to changes in testing conditions (see Additional Analyses). Having established its robustness, the results for this experiment were then analysed by fitting a mixed effects model to the performance of both groups (ISI vs no-ISI) on the SRTT. This model included *Probability* (probable vs improbable)*, Epoch* (contrasts between successive epochs 2-1, 3-2, 4-3, 5-4)*, Session* (1 vs 2) and *Group* (ISI vs no-ISI) as fixed effects and *Participants* as random effects, with a maximal-fixed-effects structure as all interactions between the predictors were analysed.

A second model was computed to explore age-related changes in procedural learning. This model included the main effects *Probability*, *Session*, *Group* and *Age* and was only estimated on the last 600 trials when procedural learning would be expected to be more salient. Since age is a continuous predictor, this variable was standardised and centred. Two participants were identified as influential for the first model and one for the second.

H2 and H3: Reliability and agreement. For split-half and test-retest reliability analysis of the SRTT were computed similarly to previous experiments, yet included Group as a predictor to account for group differences (Lammertink et al., 2020). Model random effects were extracted using the *ranef* function from the lme4 package (Bates et al., 2015).

For the PVT task, some of the most used measures were computed: lapses (number of response times equal or above 500 ms), mean RT, mean reciprocal response time (mean 1/RT) and median RT (Basner & Dinges, 2011). Additionally, an ex-Gaussian analysis was performed on the PVT for each participant to determine the levels of variability of the response times distribution, i.e. the tau parameter, whose reliability was also analysed.

H5: Explicit awareness. Explicit awareness performance was analysed by comparing the performance of each group in the generation task in both conditions (inclusion and exclusion). A two-way anova with group (ISI vs no-ISI) and condition (inclusion vs exclusion) as predictors and number of triplets recalled as an outcome variable was conducted to determine whether there were significant differences between groups in explicit awareness of the sequence at time 2 in inclusion and exclusion conditions.

Additionally, to determine whether participants presented explicit awareness beyond chance levels, chance performance was determined by randomly generating 16000 sequences of 100 keypresses which was compared to participants' scores by conducting one sample t-tests as previously done by Lee and Tomblin (2015).

H6: Relationship between procedural learning, attention, and explicit memory. Pearson correlations between procedural learning, attention and explicit were computed for each session per group. Correlations were compared using the Fisher r-to-z transformation.

Results

RT data was available for 134/135 participants for Session 1 (ISI: N = 68; no-ISI: N = 66) and for 101/135 participants for Session 2 (ISI: N = 52; no-ISI: N = 49). One participant was removed from the analysis as they had already taken part in a previous experiment, with three additional participants (ISI: N = 2; no-ISI: N = 1) being removed from analyses for Session 2 due to an administrative error. The remaining participants failed to return for Session 2. High levels of accuracy were observed across

groups and sessions (ISI: session 1 - Macc = 95%, SD = .09, session 2 - Macc = 95%, SD = .10; no-ISI: session 1 - Macc = 95%, SD = .11, session 2 - Macc = 94%, SD = .13)

H1: Procedural learning in the SRT task - Effect of ISI

A linear mixed effects regression model was fitted to the response time data. RTs decreased with practice as evidenced by the significant main effects of *Session* and *Epoch* for Epoch2-1 and Epoch4-3 (Epoch4-3 was no longer significant after correction for multiple comparisons), yet there was a group effect as the ISI group was significantly faster than the no-ISI group. Additionally, there was evidence of procedural learning - faster response times for probable than improbable trials, with this difference also increasing with practice as evidenced by a significant interaction between *Probability* and *Epoch* for Epoch2-1 and Epoch4-3 (Epoch 2-1 did not survive correction for multiple comparisons), yet there was no evidence of improvements between sessions as evidenced by the non-significant three-way interaction between *Probability*, *Epoch* and *Session*.

Despite significantly faster RTs for the ISI group, there were no significant overall group differences for procedural learning as evidenced by the two-way interaction between *Probability* and *Group*. Yet, the three-way interaction between *Probability*, *Epoch* and *Group* showed significant differences during the learning process, with the ISI group showing stronger evidence of procedural learning for the contrasts between Epoch4-3, whilst the no-ISI group showed better performance for the contrast between Epoch3-2. Only the 3-way interaction for Epoch3-2 survived correction for multiple comparisons. There was no significant difference for the first and last Epochs (Epoch 2 vs Epoch 1 and Epoch 5 vs Epoch 4). When considering the four-way interaction between *Probability*, *Epoch*, *Session* and *Group*, it showed a similar pattern to the three-way interaction, even though the only significant difference was found on the last Epoch contrast with the no-ISI group showing more evidence of procedural learning.

Figure 3

Mean and 95% CI response times for probable and improbable trials per Epoch and Session for ISI and no-ISI groups (Session 1 on the left, Session 2 on the right).

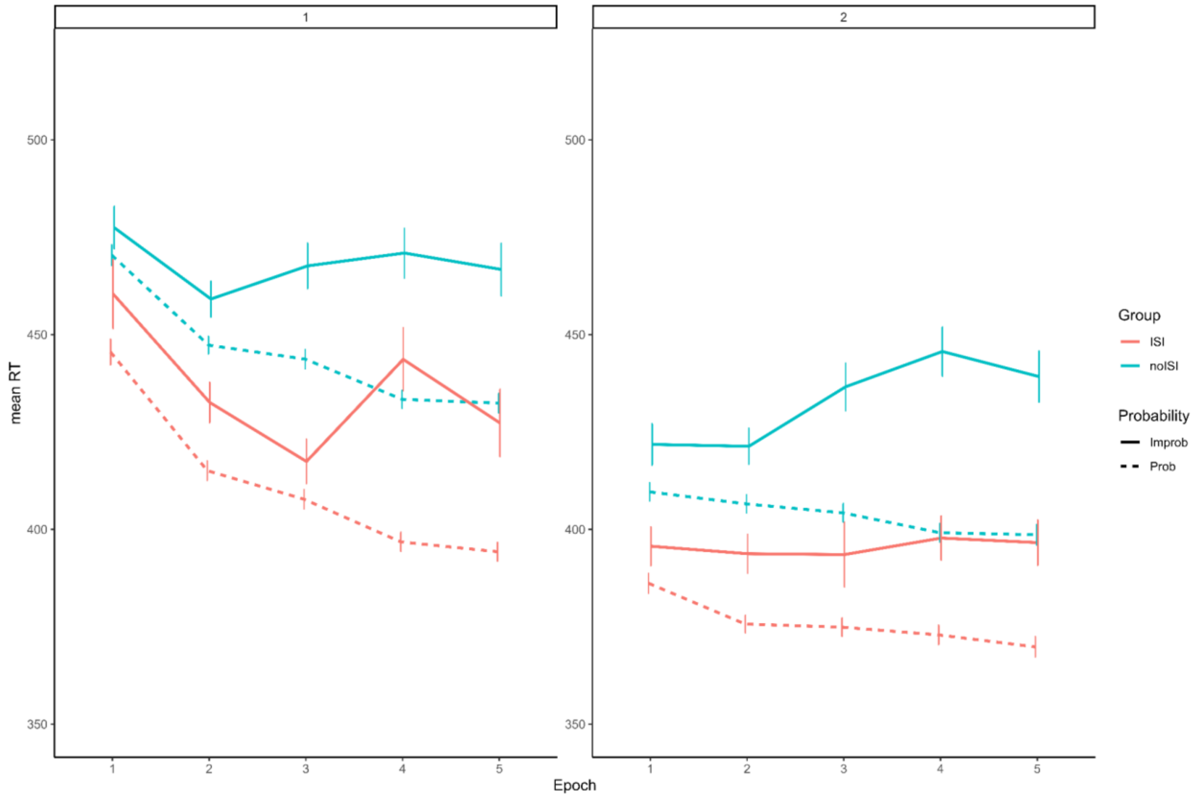

Table 3

Predictors of the magnitude of the procedural learning effect - Effect of ISI model

Fixed effects	*b*	*SE*	*t*	*p*	*CI*	
**(Intercept)**	**6.018**	**0.020**	**297.137**	**<.001**	**5.977**	**6.059**
**Probability**	**0.026**	**0.002**	**11.783**	**<.001**	**0.022**	**0.030**
**Epoch2-1**	**-0.026**	**0.006**	**-4.330**	**<.001**	**-0.038**	**-0.014**
Epoch3-2	-0.006	0.005	-1.060	.294	-0.016	0.005
Epoch4-3	0.009	0.004	2.133	.036	0.001	0.018
Epoch5-4	-0.011	0.006	-1.853	.069	-0.023	0.001
**Session**	**0.042**	**0.005**	**8.361**	**<.001**	**0.032**	**0.052**
**Group**	**-0.076**	**0.020**	**-3.750**	**.001**	**-0.117**	**-0.035**
Probability x Epoch2-1	0.008	0.003	2.732	.006	0.002	0.013
Probability x Epoch3-2	0.005	0.003	1.748	.080	-0.001	0.010
**Probability x Epoch4-3**	**0.023**	**0.003**	**7.435**	**<.001**	**0.017**	**0.029**
Probability x Epoch5-4	-0.006	0.003	-1.732	.083	-0.012	0.001

Probability x Session	-0.001	0.002	-0.862	.393	-0.005	0.002
Epoch2-1 x Session1	-0.017	0.006	-2.787	.007	-0.029	-0.005
Epoch3-2 x Session1	-0.007	0.005	-1.243	.219	-0.017	0.004
Epoch4-3 x Session1	-0.003	0.006	-0.512	.610	-0.015	0.009
Epoch5-4 x Session1	0.011	0.007	1.535	.130	-0.003	0.024
Probability x Group	0.001	0.002	0.210	.835	-0.004	0.005
Epoch2-1 x Group	0.003	0.006	0.540	.592	-0.009	0.015
Epoch3-2 x Group	-0.009	0.005	-1.665	.101	-0.019	0.002
Epoch4-3 x Group	0.002	0.004	0.337	.737	-0.007	0.010
Epoch5-4 x Group	-0.006	0.006	-1.023	.310	-0.018	0.006
Session x Group	0.003	0.005	0.533	.597	-0.007	0.013
Probability x Epoch2-1 x Session	0.003	0.003	1.066	.286	-0.002	0.008
Probability x Epoch3-2 x Session	-0.001	0.003	-0.235	.814	-0.006	0.005
Probability x Epoch4-3 x Session	0.002	0.003	0.700	.484	-0.004	0.008
Probability x Epoch5-4 x Session	-0.002	0.003	-0.659	.510	-0.008	0.004
Probability x Epoch2-1 x Group	0.001	0.003	0.248	.804	-0.005	0.006
**Probability x Epoch3-2 x Group**	**-0.010**	**0.003**	**-3.604**	**<.001**	**-0.016**	**-0.005**
Probability x Epoch4-3 x Group	0.008	0.003	2.512	.012	0.002	0.014
Probability x Epoch5-4 x Group	0.001	0.003	0.218	.828	-0.006	0.007
Probability x Session x Group	-0.001	0.002	-0.436	.665	-0.004	0.003
Epoch2-1 x Session x Group	0.002	0.006	0.284	.777	-0.011	0.014
Epoch3-2 x Session x Group	-0.001	0.005	-0.205	.838	-0.012	0.010
Epoch4-3 x Session x Group	0.000	0.006	0.063	.950	-0.012	0.012
Epoch5-4 x Session x Group	-0.010	0.007	-1.418	.162	-0.024	0.004
Probability x Epoch2-1 x Session x Group	0.000	0.003	-0.003	.998	-0.005	0.005
Probability x Epoch3-2 x Session x Group	0.000	0.003	0.043	.966	-0.005	0.006
Probability x Epoch4-3 x Session x Group	0.005	0.003	1.586	.113	-0.001	0.011

Probability x Epoch5-4 x Session x Group	-0.008	0.003	-2.534	.011	-0.014	-0.002

Random effects	*Variance*	*SD*
Participant (Intercept)	0.017	0.131
Participant: Epoch2-1	0.001	0.034
Participant: Epoch3-2	0.001	0.028
Participant: Epoch4-3	0.000	0.020
Participant: Epoch5-4	0.001	0.032
Participant: Session	0.001	0.032
Participant: Probability	0.000	0.013
Participant: Epoch2-1 x Session	0.001	0.035
Participant: Epoch3-2 x Session	0.001	0.029
Participant: Epoch4-3 x Session	0.001	0.033
Participant: Epoch5-4 x Session	0.002	0.040
Participant: Session x Probability	0.000	0.008

H4: Procedural learning in the SRT task - Age effects

To explore age differences in the performance on the SRTT, a linear mixed effects model was fitted to the RTs for the last 3 Epochs. In agreement with the previous model, RTs were significantly predicted by *Probability*. In addition to the probability effect which showed evidence for procedural learning, the two-way interaction between *Probability* x *Session* indicates that the difference between probable and improbable trials increased with experience. In line with the previous model, the procedural learning effect was similar for both groups with a non-significant interaction between *Probability* and *Group* x *Probability, Group* x *Session*. *Age* was also a significant predictor of RTs indicating that younger participants showed faster RTs than older participants (no longer significant after correction for multiple comparisons), yet there were no significant differences in procedural learning across ages as evidenced by the non-significant two-way interaction between *Probability* x *Age*, the three-way interaction between *Probability*, *Age* and *Session* and between *Probability* x *Group* x *Age* and the four-way interaction between *Probability* x *Age* x *Session* x *Group*.

Table 4

Predictors of the magnitude of procedural learning effect - Age model

Fixed effects	*b*	*SE*	*t*	*p*	*CI*	
**(Intercept)**	6.010	**0.020**	**301.991**	**<.001**	**5.970**	**6.051**
**Probability**	**0.034**	**0.003**	**12.926**	**<.001**	**0.029**	**0.040**
**Session**	**0.036**	**0.005**	**7.687**	**<.001**	**0.027**	**0.046**
**Group**	**-0.068**	**0.020**	**-3.398**	**.001**	**-0.108**	**-0.028**
Age	0.049	0.020	2.427	.019	0.008	0.090
Probability x Session	-0.002	0.002	-0.826	.413	-0.007	0.003
Probability x Group	-0.001	0.003	-0.236	.814	-0.006	0.005
Session x Group	-0.001	0.005	-0.181	.857	-0.010	0.009
Probability x Age	0.002	0.003	0.721	.475	-0.004	0.007
Session x Age	0.001	0.005	0.116	.908	-0.009	0.010
Group x Age	-0.020	0.020	-1.004	.321	-0.061	0.020
Probability x Session x Group	0.000	0.002	-0.026	.979	-0.005	0.005
Probability x Session x Age	0.001	0.002	0.558	.580	-0.003	0.006
Probability x Group x Age	0.002	0.003	0.765	.449	-0.003	0.008
Session x Group x Age	-0.013	0.005	-2.768	.008	-0.023	-0.004
Probability x Session x Group x Age	0.001	0.002	0.576	.567	-0.003	0.006

Random effects	*Variance*	*SD*
Participant (Intercept)	0.017	.129
Participant: Probability	0.001	.014
Participant: Session	0.001	.029
Participant: Probability x Session	0.000	.012

H2 and H3: Reliability and agreement

Moderate to high split-half reliability was observed for both groups, particularly when using random slopes. With the exception of the difference scores for the last 600 trials, the no-ISI group showed better split-half reliability than the ISI group. However, only the group contrasts for the random slopes in Session 1 for the whole task (Session 1: z = -4.19, p < .001) and random slopes (Session 1: z = -3.99, p < .001) remained statistically significant after correction for multiple comparisons.

Overall test-retest reliability was below psychometric standards (i.e., <.70; .01-.48). Test-retest reliability for difference scores was similar for both ISI groups. For the random slopes, the no-ISI group showed numerically higher scores when only the last 600 trials were considered (z = -2.00, p = .046) but this did not survive correction for multiple comparisons.

Following up from these analyses, we also tested for the possibility that group might have an impact on test-retest reliability by including group in a linear regression model which predicted the regression slopes in Session 2 from the random slopes in Session 1 (mean-centred), group and their interaction. There was a non-significant effect of procedural learning in session 1 on procedural learning in session 2 (overall: b = .20, SE = .13, t = 1.56, p = .123, 95% CI [-.05, .45]; last600 trials: b = .08, SE = .13, t = 0.66, p = .513, 95% CI [-0.17, 0.34]). However, we found evidence that group moderated the relationship between procedural learning Sessions 1 and 2 (overall: b = -.34, SE = .20, t = -1.72, p = .089, 95% CI [-.727, .053]; last600 trials: b = -.50, SE = .19, t = -2.59, p = .011, 95% CI [-.89, -.12]) and a significant interaction between group and the procedural learning effect in session 1, suggesting that group membership (i.e., performing the SRT with or without a 250ms ISI) influenced the test-retest reliability of the SRTT. However, both results were only significant for the last 600 trials (overall: b = .25, SE = .20, t = 1.25, p = .216, 95% CI [-.148, .646]; last600 trials: b = .44, SE = .20, t = 2.16, p = .033, 95% CI [.04, .85]).

Table 5

Split-half and test-retest reliability of the procedural learning measures for overall and last 600 trials of the SRTT for session 1 and session 2

Measure		Split-half reliability				Test-retest reliability	
		ISI		no ISI		ISI	no ISI
		Session 1 (N 62 – 65)	Session 2 (N 48 – 49)	Session 1 (N 59 – 61)	Session 2 (N 45 – 45)	Session 1-2 (N 49 – 50)	Session 1-2 (N 43 – 45)
Difference	Overall	.56 (.37, .71)	.69 (.50, .81)	.67 (.50, .79)	.77 (.62, .87)	.19 (-.09, .45)	.08 (-.23, .36)
	Last 600 trials	.53 (.32, .69)	.58 (.36, .74)	.52 (.31, .69)	.60 (.38, .76)	.01 (-.27, .29)	.03 (-.28, .32)
Random Slopes	Overall	.71 (.56, .81)	.69 (.51, .81)	.93 (.88, .96)	.83 (.71, .90)	.20 (-.08, .46)	.44 (.17, .65)
	Last 600 trials	.72 (.58, .82)	.58 (.36, .74)	.93 (.88, .95)	.74 (.57, .85)	.09 (-.19, .36)	.48 (.21, .68)

Bland-Altman plots were used to compare the agreement between procedural learning for the last 3 epochs for each group. These revealed slightly smaller limits of agreement (lower and upper black lines in Figure 2) for the no-ISI group (-53.43; 48.76) than the ISI-group (-55.33; 55.24), indicating a more consistent performance for individuals in the former group. Irrespective of the previous differences, both groups showed wide limits of agreement, suggesting that there is an unacceptable degree of agreement.

Figure 4

Plot of the mean of the two measurements against the differences between procedural learning in session 1 and session 2 for the ISI (A) and no-ISI (B) groups

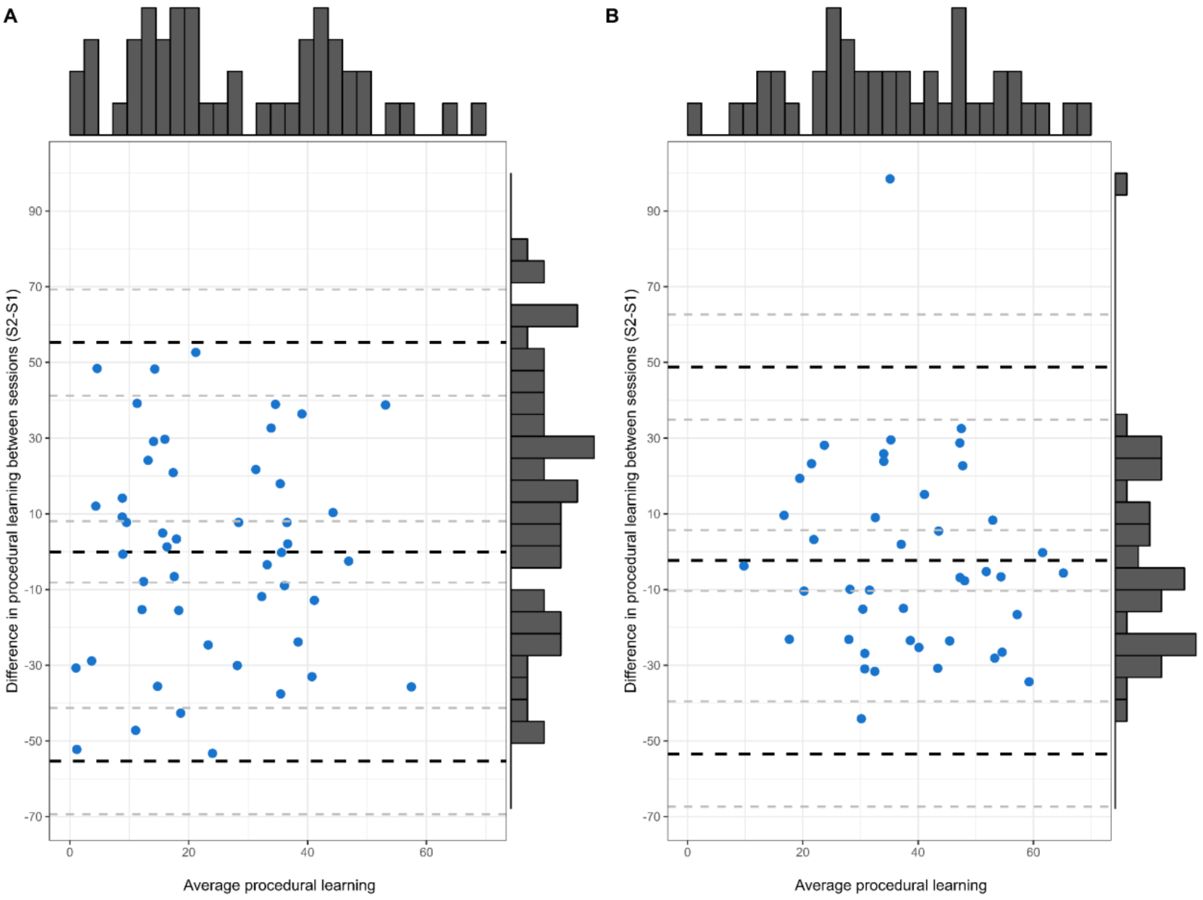

Split-half reliability for the Psychomotor Vigilance task varied between .32 to .90 with similar scores for both sessions, except for mean RTs. Test-retest reliability ranged from .51 to .76. Median RTs shows the highest split-half (*r* ≥.90) and test-retest reliability (*r* = .76) at both time points. Mean RTs showed the lowest split-half and test-retest reliability of all measures.

Table 6

Descriptive statistics, split-half and test-retest reliability of the psychomotor vigilance task

PVT measure	Descriptive statistics						Split-half reliability		Test-retest reliability
	Session 1			Session 2			Session 1	Session 2	
	N	M	SD	N	M	SD			

Lapses	97	1.79	2.34	62	2.16	3.15	.50[3] (.33, .63)	.49[3] (.27, .67)	.53[3] (.31, .70)
Mean RTs	99	338.59	88.81	63	353.58	80.83	.77 (.68, .84)	.32 (.08, .52)	.51 (.28, .68)
Median	99	306.35	53.68	63	323.83	50.00	.93 (.89, .95)	.90 (.84, .94)	.76 (.63, .86)
Reciprocal	97	3.43	1.33	64	3.16	.52	.65 (.52, .76)	.84 (.74, .90)	.60 (.40, .74)

Ex-gaussian

Similarly to the findings of experiment 2, there was a pattern for a numerically higher reliability for the procedural learning effect for those who showed lower variability on the PVT task (lower tau), but only on the second session. Yet, this effect only emerged for the no-ISI group (comparable to the conditions in experiment 2).

Table 7

Test-retest reliability for low and high tau groups for each session

Session	ISI		no-ISI	
	low-tau	high-tau	low-tau	high-tau
1	$r(18) = .11$ (-.37, .55)	$r(22) = .03$ (-.40, .44)	$r(19) = .51$* (.08, .78)	$r(17) = .47$† (-.02, .77)
2	$r(15) = -.06$ (-.55, .47)	$r(13) = -.05$ (-.59, .51)	$r(15) = .62$* (.15, .86)	$r(15) = .30$ (-.25, .71)

Note. †$p < .10$; *$p < .05$

[3] The test-retest and split-half reliability for the number of lapses was computed using a Spearman correlation as recommended by Green (2021) for zero-inflated variables.

H5: Explicit awareness

Each generated sequence was coded on the number of triplets recalled in common with the training sequence with a maximum score of 98 triplets. Chance level performance was estimated to be 29.98 triplets.

Table 8

Descriptive statistics (means and standard deviations) for the explicit awareness tasks

**TRIPLETS**	**ISI**			**no-ISI**		
	N	Mean	SD	N	Mean	SD
**Inclusion**	36	36.92	6.23	38	36.89	9.27
**Exclusion**	37	28.62	14.80	40	28.55	11.90

Both groups showed a similar performance on the explicit awareness tasks (Table 8) as evidenced in the two-way ANOVA, with *Group* as a non-significant predictor of performance on these tasks ($F(1, 144) = .001$, $p = .98$). There was a statistically significant effect of *Condition* (inclusion vs exclusion; $F(1, 144) = 20.95$, $p = <.001$), as participants recalled more triplets in the inclusion than in the exclusion condition, but no significant interaction between *Group* x *Condition* ($F(1, 144) = .00$, $p = .99$).

Comparisons against chance level for the inclusion task revealed a statistically significant higher number of triplets recalled by participants when compared to chance level for both ISI ($t(35) = 6.68$, $p < .001$) and no-ISI groups ($t(36) = 4.54$, $p < .001$). For the exclusion task, participants in both groups performed similarly to chance levels (ISI: $t(36) = -.56$, $p = .581$; no-ISI: $t(37) = -.74$, $p = .466$), thus revealing some control over their explicit knowledge of the sequence. Thus, contrary to predictions, the ISI condition did not result in greater levels of explicit awareness when compared to the no-ISI condition.

Figure 5

Violin and boxplots showing the distribution of number of items recalled in the generation tasks in the inclusion and exclusion conditions by group

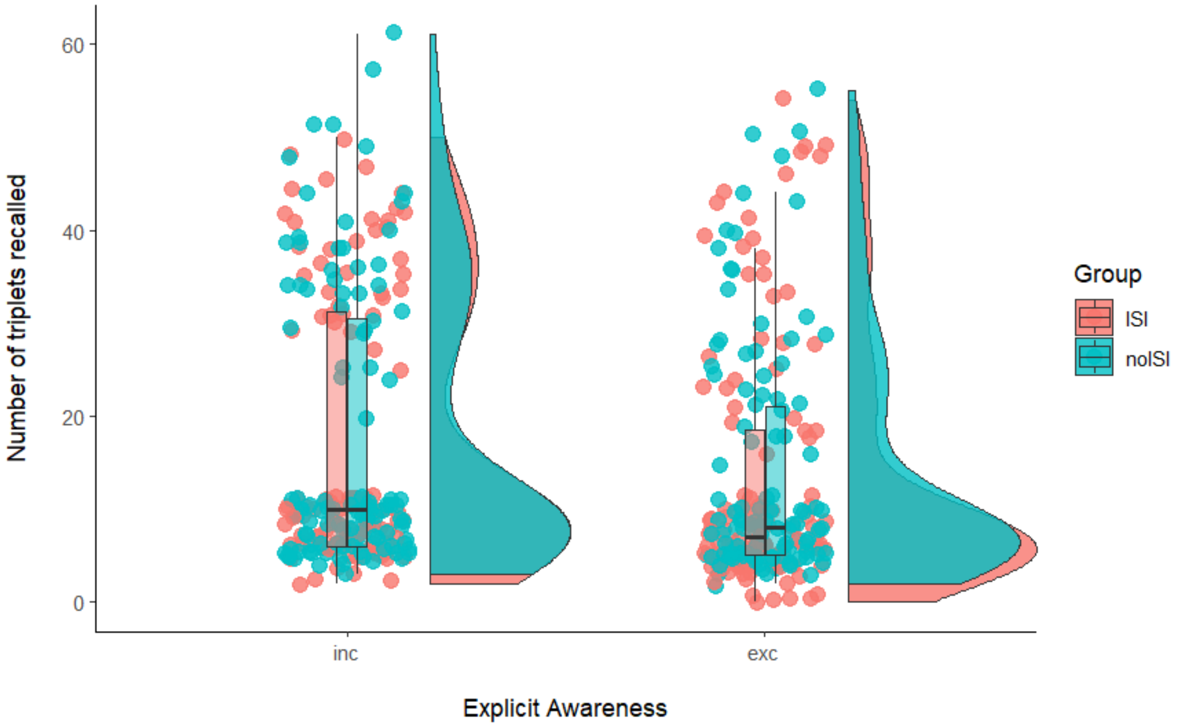

H5 and H6: Relationship between procedural learning and attention, and explicit awareness

Pearson correlations revealed a significant negative association between attention on both sessions and procedural learning for the ISI group for session 1 and 2 (Session 1: r = -.42, Session 2: -.38), yet only the association for Session 1 remained significant after correction. This association indicates that participants with better attention show more evidence of procedural learning when the sequence is presented more slowly. None of the other correlations between the performance on the PVT and procedural learning were significant for either the ISI or no-ISI groups. Similarly, there were no significant correlations between explicit awareness and procedural learning ($p > .05$). None of the group contrasts were statistically significant ($p > .05$).

Table 9

Pearson correlations between procedural learning in the SRTT and attention and explicit awareness measures

**CORRELATIONS**	**ISI**		**no-ISI**	
	SRT1	SRT2	SRT1	SRT2

	N	r	N	r	N	r	N	r
Median 1	50	**-.42*** **	41	.12	45	-.02	37	-.23
Median 2	30	-.38*	27	.09	34	.23	31	-.32
Inclusion generation	36	-.16	35	-.03	38	.18	36	.09
Explicit generation	36	.10	35	.03	39	.14	37	-.19

Note: ***p<.001; * p<.05; bold – survived corrections for multiple comparisons using the Holm-Bonferroni Method (Holm, 1979).

Discussion

As in Experiments 1 and 2, there was clear evidence of procedural learning for both groups and Sessions. The ISI group showed faster response times than the no-ISI group as expected (Martini et al., 2013) but this did not translate into differences in procedural learning. However, there was a small, and non-significant, increase in the procedural learning effect from session 1 to session 2 for the no-ISI group whilst the opposite was observed for the ISI group. Split-half reliability was again higher than stability across sessions for both groups, with significantly better split-half reliability for the no-ISI group. Test-retest reliability was also again below psychometric standards and did not differ statistically for the no-ISI and ISI groups (r = .42 and r = .21, respectively). Thus, counter to predictions, including an ISI did not lead to improved stability; in fact, there was more evidence for the reverse pattern. Therefore, the presence of an ISI cannot account for the higher levels of stability reported by West et al. (2021). In the current experiment, the ISI group showed lower levels of procedural learning overall, as well as higher within-subject variability, than those reported by West et al. (2021) (Session 1: M = 96.06; SD = 69.73; Session 2: M = 103.33; SD = 55.02). This may be because the ISI task characteristics may have led to increased attentional demands for the ISI group given that the duration of the ISI version of the SRTT lasted approximately 4 minutes longer (representing a 33% increase in task duration) than the no-ISI version. Corroborating this hypothesis there was evidence of a stronger correlation between attention and procedural learning and higher response time variability for this group (ISI: 399.83 (132.59); no-ISI: 431.65 (118.48)). Thus, fluctuations in attention and decreases in motivation would be more likely to occur for the ISI version, which is in line with the poor enjoyment of the task observed by West et al. (2021).

The finding of a significant association between attention and procedural learning for the ISI group only conflicts with Experiment 2, where an association was observed with a 0ms ISI. However,

this could be due to the use of the 5-minute PVT (as opposed to the 10-minute task used in Experiment 2). Indeed, a follow-up experiment (Additional Analyses 9) replicated the correlation between procedural learning and attention for a no-ISI group (N = 52) when using the 10-minute version online.

Counter to predictions there was no effect of age on procedural learning. Similarly, ISI groups showed comparable performance on the explicit awareness tasks (counter to Huang et al., 2017; Verneau et al., 2014) and there were no associations between explicit awareness and procedural learning.

In sum, both ISI groups showed comparable procedural learning effects, and there was no evidence that the ISI group showed greater reliability. In fact, the no-ISI group showed greater within-session reliability than the ISI group. Similarly, there was no effect of age on procedural learning or reliability. Thus, neither ISI nor age account for the higher retest reliability previously reported by West et al. (2021). Finally, Experiment 2 examines the use of the SRTT in an online setting, importantly demonstrating that the magnitude and trajectory of procedural learning, as well as its stability, is consistent with data collected in the lab. This suggests that, despite its poor psychometric properties, this task can reveal robust procedural learning effects in these different settings.

References

Arnon, I. (2019). Statistical Learning, Implicit Learning, and First Language Acquisition: A Critical Evaluation of Two Developmental Predictions. *Topics in Cognitive Science*, tops.12428. https://doi.org/10.1111/tops.12428

Basner, M., & Dinges, D. F. (2011). Maximizing sensitivity of PVT to Sleep Loss (Basner, Dinges). *Sleep*, *34*(5), 581–591.

Bates, D., Mächler, M., Bolker, B., & Walker, S. (2015). Fitting Linear Mixed-Effects Models Using lme4. *Journal of Statistical Software*, *67*(1), 1–48. https://doi.org/10.18637/jss.v067.i01

Bridges, D., Pitiot, A., MacAskill, M. R., & Peirce, J. W. (2020). The timing mega-study: Comparing a range of experiment generators, both lab-based and online. *PeerJ*, *8*, e9414–e9414. https://doi.org/10.7717/peerj.9414

Destrebecqz, A., & Cleeremans, A. (2001). Can sequence learning be implicit? New evidence with the process dissociation procedure. *Psychonomic Bulletin & Review*, *8*(2), 343–350. https://doi.org/10.3758/BF03196171

Destrebecqz, A., & Cleeremans, A. (2003). *Temporal effects in sequence learning*. 181–213. https://doi.org/10.1075/aicr.48.11des

Farkas, B., Krajcsi, A., Janacsek, K., & Nemeth, D. (2023). *The complexity of measuring reliability in learning tasks: An illustration using the Alternating Serial Reaction Time task*. https://doi.org/10.3758/s13428-022-02038-5

Franklin, M. S., Smallwood, J., Zedelius, C. M., Broadway, J. M., & Schooler, J. W. (2016). Unaware yet reliant on attention: Experience sampling reveals that mind-wandering impedes implicit learning. *Psychonomic Bulletin and Review*, *23*(1), 223–229. https://doi.org/10.3758/s13423-015-0885-5

Green, J. (2021). Too many zeros and/or highly skewed? A tutorial on modelling health behaviour as count data with Poisson and negative binomial regression. *Health Psychology and Behavioral Medicine*, *9*, 436–455.

https://doi.org/10.1080/21642850.2021.1920416

Hedge, C., Powell, G., & Sumner, P. (2018). The reliability paradox: Why robust cognitive tasks do not produce reliable individual differences. *Behavior Research Methods*, *50*(3), 1166–1186. https://doi.org/10.3758/s13428-017-0935-1

Holm, S. (1979). A simple sequentially rejective multiple test procedure. *Scandinavian Journal of Statistics*, *6*(2), 65–70.

Huang, J., Li, Y., Zhang, J., Wang, X., Huang, C., Chen, A., & Liu, D. (2017). FMRI Investigation on Gradual Change of Awareness States in Implicit Sequence Learning. *Scientific Reports*, *7*(1), 16731. https://doi.org/10.1038/s41598-017-16340-2

Jimenez, L., & Mendez, C. (1999). Which Attention Is Needed for Implicit Sequence Learning?? *Experimental Psychology: Learning, Memory, and Cognition*, *25*(1), 236–259. https://doi.org/10.1037/0278-7393.25.1.236

Lammertink, I., Boersma, P., Wijnen, F., & Rispens, J. (2020). Statistical Learning in the Visuomotor Domain and Its Relation to Grammatical Proficiency in Children with and without Developmental Language Disorder: A Conceptual Replication and Meta-Analysis. *Language Learning and Development*, *16*(4), 426–450. https://doi.org/10.1080/15475441.2020.1820340

Lee, J. C., & Tomblin, J. B. (2015). Procedural Learning and Individual Differences in Language. *Language Learning and Development*, *11*(3), 215–236. https://doi.org/10.1080/15475441.2014.904168

Martini, M., Furtner, M. R., & Sachse, P. (2013). Working Memory and Its Relation to Deterministic Sequence Learning. *PLoS ONE*, *8*(2). https://doi.org/10.1371/journal.pone.0056166

Peirce, J., Gray, J. R., Simpson, S., MacAskill, M., Höchenberger, R., Sogo, H., Kastman, E., & Lindeløv, J. K. (2019). PsychoPy2: Experiments in behavior made easy. *Behavior Research Methods*, *51*(1), 195–203. https://doi.org/10.3758/s13428-018-01193-y

Reifman, J., Kumar, K., Khitrov, M. Y., Liu, J., & Ramakrishnan, S. (2018). PC-PVT 2.0: An

updated platform for psychomotor vigilance task testing, analysis, prediction, and visualization. *Journal of Neuroscience Methods*, *304*, 39–45. https://doi.org/10.1016/j.jneumeth.2018.04.007

Seger, C. A., & Spiering, B. J. (2011). A Critical Review of Habit Learning and the Basal Ganglia. *Frontiers in Systems Neuroscience*, *5*(August), 1–9. https://doi.org/10.3389/fnsys.2011.00066

Shanks, D. R., Rowland, L. A., & Ranger, M. S. (2005). Attentional load and implicit sequence learning. *Psychological Research*, *69*(5–6), 369–382. https://doi.org/10.1007/s00426-004-0211-8

Song, S., Howard, J. H., & Howard, D. V. (2007). Implicit probabilistic sequence learning is independent of explicit awareness. *Learning & Memory*, *14*(3), 167–176. https://doi.org/10.1101/lm.437407

Spearman, C. (1910). Correlation calculated from faulty data. *British Journal of Psychology, 1904-1920*, *3*(3), 271–295. https://doi.org/10.1111/j.2044-8295.1910.tb00206.x

Verneau, M., Van Der Kamp, J., Savelsbergh, G. J. P., & De Looze, M. P. (2014). Age and time effects on implicit and explicit learning. *Experimental Aging Research*, *40*(4), 477–511. https://doi.org/10.1080/0361073X.2014.926778

Wagner, R. K., Torgesen, J. K., Rashotte, C. A., & Pearson, N. A. (2013). *CTOPP-2 Comprehensive Test of Phonological Processing – Second Edition.* Pearson Clinical.

Wechsler, D. (2009). *Wechsler Individual Achievement Test – Third UK Edition (WIAT-III UK)*. Pearson Assessment.

Wechsler, D. (2011). *Wechsler Abbreviated Scale of Intelligence – Second Edition (WASI-II)*. NCS Pearson.

West, G., Shanks, D. R., & Hulme, C. (2021). Sustained Attention, Not Procedural Learning, is a Predictor of Reading, Language and Arithmetic Skills in Children. *Scientific Studies of Reading*, *25*(1), 47–63. https://doi.org/10.1080/10888438.2020.1750618

Wiig, E. H., Semel, E., & Secord, W. A. (2013). *Clinical Evaluation of Language Fundamentals—Fifth UK Edition*. Pearson Assessment.

Wilkinson, L., & Shanks, D. R. (2004). Intentional Control and Implicit Sequence Learning. *Journal of Experimental Psychology: Learning, Memory, and Cognition*, *30*(2), 354–369. https://doi.org/10.1037/0278-7393.30.2.354
